# Enhanced liquidity of p62 droplets mediated by Smurf1 links Nrf2 activation and autophagy

**DOI:** 10.1186/s13578-023-00978-9

**Published:** 2023-02-21

**Authors:** Qin Xia, Yang Li, Wanting Xu, Chengwei Wu, Hanfei Zheng, Liqun Liu, Lei Dong

**Affiliations:** School of Life Science, Beijing Institute of Technology, No. 5, South Street, Zhongguancun, Haidian District, Beijing, China

**Keywords:** Smurf1, Protein homeostasis, p62-liquid droplets

## Abstract

**Background:**

Macro-autophagy/Autophagy is an evolutionarily well-conserved recycling process to maintain the balance through precise spatiotemporal regulation. However, the regulatory mechanisms of biomolecular condensates by the key adaptor protein p62 via liquid-liquid phase separation (LLPS) remain obscure.

**Results:**

In this study, we showed that E3 ligase Smurf1 enhanced Nrf2 activation and promoted autophagy by increasing p62 phase separation capability. Specifically, the Smurf1/p62 interaction improved the formation and material exchange of liquid droplets compared with p62 single puncta. Additionally, Smurf1 promoted the competitive binding of p62 with Keap1 to increase Nrf2 nuclear translocation in p62 Ser349 phosphorylation-dependent manner. Mechanistically, overexpressed Smurf1 increased the activation of mTORC1 (mechanistic target of rapamycin complex 1), in turn leading to p62 Ser349 phosphorylation. Nrf2 activation increased the mRNA levels of Smurf1, p62, and NBR1, further promoting the droplet liquidity to enhance oxidative stress response. Importantly, we showed that Smurf1 maintained cellular homeostasis by promoting cargo degradation through the p62/LC3 autophagic pathway.

**Conclusions:**

These findings revealed the complex interconnected role among Smurf1, p62/Nrf2/NBR1, and p62/LC3 axis in determining Nrf2 activation and subsequent clearance of condensates through LLPS mechanism.

**Supplementary Information:**

The online version contains supplementary material available at 10.1186/s13578-023-00978-9.

## Introduction

The biological macromolecules, such as proteins and nucleic acids, spontaneously transition to liquid droplets [also referred to as membrane-less organelles, formed by liquid-liquid phase separation (LLPS)] to maintain homeostasis by controlling nuclear function, regulating cellular quality control, and organizing biochemical networks [1]. They display liquid-like properties, such as highly dynamic interiors capable of rapid rearrangement and stochastic exchange with the bulk solution, as well as dynamic fusion, deformation, and fission [2–5]. The condensates change from liquid droplets to gel-like or solid-like materials with increased interaction density between molecules [6]. The abnormal regulation of liquid droplets is the pathogenesis of multiple neurodegenerative disorders, such as Alzheimer’s disease, Parkinson’s disease, amyotrophic lateral sclerosis, and so forth [7–10]. Importantly, dysregulated LLPS is increasingly implicated as a previously hidden driver of oncogenic activity as follows. (1) The gain-of-function mutation of tumor suppressor p53 spontaneously transitions into amyloid oligomers (active state; reversible) or larger aggregates (solid-state; less reversible) to promote oncogenesis [11]. (2) The phase separation of metastasis-promoting transcriptional factor TAZ (transcriptional coactivator with PDZ-binding motif) compartmentalized the machinery to promote tumorigenesis [12]. (3) Gain-of-function mutant Src homology-2 domain-containing protein tyrosine phosphatase-2 (SHP2) initiates mitogen-activated protein kinases (MAPK) signalling and activation of RAS signalling to promote tumorigenesis by increasing the formation of biomolecular condensates [13, 14]. (4) A mutation in E3 ligase adaptor SPOP (speckle type BTB/POZ protein) promotes the initiation and progression of prostate cancer through SQSTM1(Sequestosome-1)/p62-dependent autophagy and Nrf2(nuclear factor erythroid-2 related factor 2) activation [15].

The ubiquitin-proteasome system (UPS) and autophagy are the main cellular clearing systems to maintain protein homeostasis in response to extreme environmental stress (e.g., proteotoxic and oxidative stresses) [16]. The p62-dependent compartmentalization is an interconnected quality control system by decision-making based on the binding affinities, LLPS, or formation of aggregates [17]. The p62 binds to and condensates the ubiquitinated proteins for autophagic degradation to compensate for UPS overload or deficiency [18]. Recently, the liquidity allowing the exchange of the components with the surrounding environment has been associated with selective autophagy [5, 19]. The p62 mediates the phase separation of ubiquitinated proteins to form condensates [5]. The p62 gel-like condensates interact with autophagy-initiating protein FAK family kinase-interacting protein of 200 kDa (FIP200) to drive the formation of a large-size phagophore, which is degraded by autophagy [20]. Moreover, the p62 liquid droplet was found to enhance oxidative stress response through activating Nrf2 [21, 22]. Keap1 interacts with Nrf2 and promotes its degradation [23]. Studies revealed that p62 gels interfered with Keap1-Nrf2 interaction by competitively binding with Keap1, leading to the Nrf2 nuclear translocation and upregulating the expression of antioxidant response elements containing genes such as *HO1* (*Heme Oxygenase 1*), *NQO1*(NAD(P)H Quinone Dehydrogenase 1)*,* and *p62* [23]. Especially, p62-mediated Nrf2 activation triggered by another adaptor protein NBR1 (neighbor of BRCA1 gene 1), a functional analog of p62, further promotes phase separation and phosphorylation of p62 [18, 24]. However, the regulatory mechanisms of p62 condensate-dependent Nrf2 activation in response to stress remain obscure.

Oncogenic E3 ligase SMAD Specific E3 Ubiquitin Protein Ligase 1 (Smurf1) is highly expressed in cancer cells and recognized as a new mediator of selective autophagy by interacting with the nascent autophagosome membrane [25–27]. This study further found that Smurf1 increased and co-localized with p62 droplets in response to oxidant stress by recruiting LC3, Keap1, and another autophagy adaptor NBR1. It indicated that Smurf1 promoted the formation and material exchange of droplets through the phase separation of p62. This study underscored that Smurf1 promoted tumor cell survival by upregulating p62 liquid droplet formation and degradation and might serve as a potential target for cancer therapy.

## Results

### Smurf1 promotes the formation of p62-liquid droplets

**Fig. 1:**
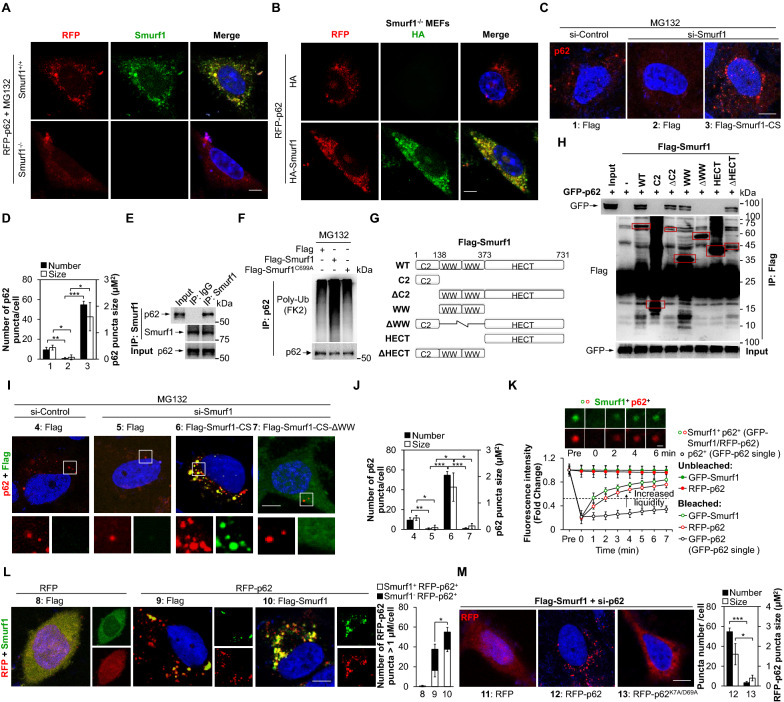
Smurf1 promotes the formation of p62-liquid droplets. **A** The *Smurf1*^+/+^ and *Smurf1*^−/−^ MEF cells were transfected with RFP-p62, and fixed after treating with MG132 (20 µM, 12 h), and then immunofluorescence stained with anti-Smurf1 antibody. The nucleus was stained by DAPI. Bar: 5 µm. **B** The *Smurf1*^−/−^ MEF cells were transfected with RFP-p62 and fixed after transfection with HA or HA-Smurf1, and then immunofluorescence stained with anti-HA antibody. Bar: 5 µm. The nucleus was stained by DAPI. **C**, **D** LN229 cells treated with control or Smurf1 siRNA oligos were transfected with Flag or Flag-Smurf1-CS, fixed after treating with MG132 (20 µM, 12 h) and then performed by immunofluorescence analysis with anti-p62 antibody. The nucleus was stained with DAPI. Bar: 5 µm (**C**). Bar graphs indicate the number and size of p62 puncta in each cell (mean ± SD, n = 10 cells examined over three independent experiments). * p < 0.05, ** p < 0.01, *** p < 0.001 as determined by unpaired two-tailed Student’s *t*-test (**D**). **E** 293T cells lysates were immunoprecipitated by IgG or Smurf1 antibody. The immunoprecipitates and the input were probed with anti-p62 and anti-Smurf1 antibodies. **F** 293T cells were transfected with Flag, Flag-Smurf1 or Flag-Smurf1^C699A^. Cell lysates were immunoprecipitated by anti-p62 antibody. The immunoprecipitates were probed with anti-Ub and anti-p62 antibodies. **G**, **H** 293T cells with GFP-p62 overexpression were transfected with Flag, Flag-Smurf1, Flag-Smurf1-C2, Flag-Smurf1-ΔC2, Flag-Smurf1-WW, Flag-Smurf1-ΔWW, Flag-Smurf1-HECT or Flag-Smurf1-ΔHECT (G, schematic diagram). Cell lysates were immunoprecipitated by anti-GFP antibody. The immunoprecipitates and the input were probed with anti-GFP and anti-Flag antibodies (H). **I**, **J** LN229 cells treated with control or Smurf1 siRNA oligos were transfected with Flag, Flag-Smurf1-CS, or Flag-Smurf1-CS-ΔWW, fixed after treating with MG132 (20 µM, 12 h) and then performed by immunofluorescence analysis with anti-p62 and anti-Flag antibody. The nucleus was stained with DAPI. Bar: 5 µm (**I**). Bar graphs indicate the number and size of p62 puncta in each cell (mean ± SD, n = 10 cells examined over three independent experiments). * p < 0.05, ** p < 0.01, *** p < 0.001 as determined by unpaired two-tailed Student’s t-test (**J**). **K** LN229 cells cultured in glass-bottom plates were transfected with GFP-p62 alone or co-transfected with GFP-Smurf1 or RFP-p62. Bar: 1 µm. The signal recovery after photobleaching was measured by Image J; mean ± SD, n = 20 droplets examined over three independent experiments. **L** LN229 cells with RFP or RFP-p62 overexpression were transfected with Flag or Flag-Smurf1, fixed, and then immunofluorescence stained with anti-Smurf1 antibody. The nucleus was stained with DAPI. Bar: 5 µm. The number of RFP-p62 puncta > 1 µm in each cell was assessed (mean ± SD, n = 10 cells examined over three independent experiments). * p < 0.05 as determined by unpaired two-tailed Student’s t-test. **M** LN229 cells treated with p62 siRNA oligos were transfected with Flag-Smurf1, then fixed after overexpressing RFP, RFP-p62, or RFP-p62^K7A/D69A^. Bar: 5 µm. Bar graphs indicate the number and size of RFP-p62 puncta in each cell (mean ± SD, n = 10 cells examined over three independent experiments). The nucleus was stained with DAPI. * p < 0.05, *** p < 0.001 as determined by unpaired two-tailed Student’s *t*-test

To confirm the effect of Smurf1 on p62 puncta formation under stress, we first treated Smurf1^KO^ (*Smurf1*^−/−^) and Wild-type (*Smurf1*^+/+^) cells with a proteasome inhibitor MG132. The mouse embryonic fibroblasts (MEFs) were generated from *Smurf1*^−/−^ and *Smurf1*^+*/*+^ mice. We found more RFP-p62 puncta in *Smurf1*^+*/*+^ MEFs than *Smurf1*^*−/−*^ MEFs, under MG132 treatment (Fig. 1A), indicating Smurf1 deficiency blocked the p62 puncta formation. Then we transfected HA or HA-Smurf1 into *Smurf1*^−/−^ MEFs with RFP-p62, and found that Smurf1 overexpression also increased the number of RFP-p62 puncta in *Smurf1*^*−/−*^ MEFs (Fig. 1B). To further evaluate the role of Smurf1 in the formation of p62 puncta, we knocked down or overexpressed Smurf1 in glioblastoma LN229 cells. We found that Flag-Smurf1 failed to increase the number of p62 puncta under basal condition (Additional file 1: Fig. S1A), indicating stress is the initial trigger factor in the p62 aggregation. Importantly, the number and the size of p62 puncta were decreased by si-Smurf1 and recovered by overexpression of Flag-Smurf1-CS (codon switch, si-Smurf1 resistant) under MG132 treatment (Fig. 1C, D).

To test whether the E3 ligase activity of Smurf1 promote p62 puncta formation under stress, we overexpressed Flag-Smurf1 or Flag-Smurf1^C699A^ (E3 activity deficiency) in LN229 cells. The number and size of p62 puncta failed to respond to Flag-Smurf1^C699A^ under MG132 treatment compared with Flag-Smurf1, suggesting Smurf1 promoted the formation of p62 puncta in its E3 ligase activity-dependent manner (Additional file 1: Fig. S1B). Moreover, we found endogenous Smurf1 interacts or colocalizes with p62 (Fig. 1E). Flag-Smurf1, but not Flag-Smurf1^C699A^, promoted the binding of p62 to ubiquitin under MG132 treatment, suggesting that Smurf1 may increase the p62-ubiquitin binding in its E3 ligase activity-dependent manner (Fig. 1F). To test which domain of Smurf1 could interact with p62, we overexpressed GFP-p62 in LN229 cells with Flag-Smurf1 or Flag control. Immunoprecipitation analysis found that Flag-Smurf1, Flag-Smurf1-ΔC2, Flag-Smurf1-WW, and Flag-Smurf1-ΔHECT (but not Flag, Flag-Smurf1-C2, Flag-Smurf1-ΔWW and Flag-Smurf1-HECT) interacted with GFP-p62 (Fig. 1G, H). The number and size of p62 puncta failed to respond to Flag-Smurf1-CS-ΔWW under MG132 treatment compared with Flag-Smurf1-CS, suggesting Smurf1-WW interact with p62 to increase liquid droplets formation (Fig. 1I, H).

Previously, the p62 droplets are subject to liquidity characterized by fluorescence recovery after photobleaching (FRAP) assay [2, 5]. To determine if Smurf1 promotes liquidity of p62, we overexpressed fluorescence-labeled p62 and Smurf1 in glioblastoma cells. We found that Smurf1 promoted the capability of fluorescence recovery of double-positive puncta, evidenced by the half-recovery time after photobleaching (*t*1/2) of RFP-p62 in GFP-Smurf1^+^/RFP-p62^+^ puncta were generally at 2 ± 1 min compared to GFP-p62 single puncta at 12 ± 3 min, indicating that Smurf1 facilitate p62 to induce exchanging molecules with the surrounding environment (Fig. 1K and Additional file 1: Fig. S1C). In addition, we found that Smurf1^+^ puncta also displayed liquid properties, and the *t*1/2 of GFP-Smurf1 single in GFP-Smurf1^+^RFP-p62^+^ puncta (1 ± 0.5 min) was shorter than GFP-Smurf1 single puncta (3 ± 1 min) (Fig. 1K and Additional file 1: Fig. S1D). Flag-Smurf1 overexpression indeed co-localized with p62 and increased the number of Smurf1^+^p62^+^ (including endogenous Smurf1 and Flag-Smurf1) puncta, suggesting that Smurf1-induced exchanging molecules may depend on p62 phase separation ability (Fig. 1L). The K7A/D69A double-mutation disrupts the oligomerization and phase separation of p62 [18, 20, 28, 29]. We knocked down p62, then overexpressed p62^WT^ and a phase separation‐defective p62^K7A/D69A^ plasmid, and found that RFP-p62^WT^ droplets, but not RFP-p62^K7A/D69A^ droplets, formed in Flag-Smurf1 LN229 cells, suggesting Smurf1-induced formation of p62 liquid droplets is dependent on the p62 phase separation ability (Fig. 1M).

## p62 phase separation is required for Smurf1-mediated Nrf2 activation

**Fig. 2:**
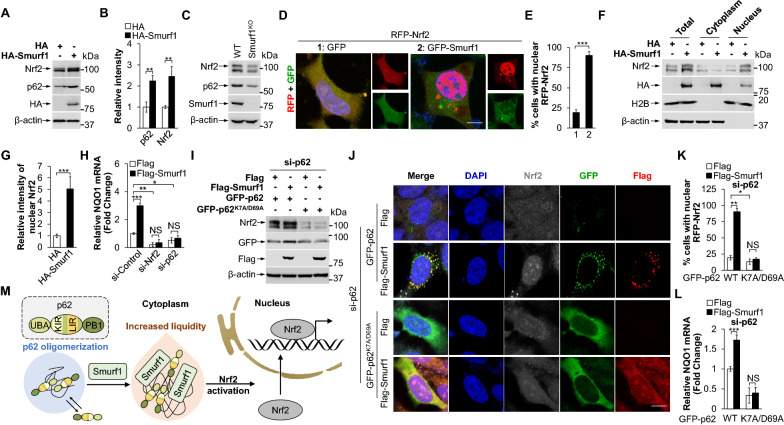
p62 phase separation is required for Smurf1-mediated Nrf2 activation. **A**, **B** LN229 cells were transfected with HA or HA-Smurf1. Cell lysates were prepared and subjected to western blot analysis with the indicated antibodies (anti-Nrf2, anti-p62, anti-HA, and anti-β-actin) (**A**). The panels show relative intensity of p62 and Nrf2 in total cell (mean ± SD from 3 independent experiments). ** p < 0.01 as determined by unpaired two-tailed Student’s *t*-test (**B**). **C** The MEF cells were isolated from *Smurf1*^+/+^ (WT) and *Smurf1*^−/−^ (Smurf1^KO^)mice. Cell lysates were prepared and subjected to western blot analysis with the indicated antibodies (anti-Nrf2, anti-p62, anti-Smurf1, and anti-β-actin). **D**, **E** LN229 cells overexpressing RFP-Nrf2 were fixed after transfecting with GFP or GFP-Smurf1. The nucleus was stained with DAPI. Bar: 5 µm (**D**). More than 100 cells were assayed for the nuclear signal of Nrf2; mean ± SD from 3 independent experiments. *** p < 0.001 as determined by unpaired two-tailed Student’s *t*-test (**E**). **F**, **G** 293T cells were transfected with HA or HA-Smurf1. Cytosolic and nuclear fractions of cells were separated and subjected to western blot analysis with the indicated antibodies (anti-Nrf2, anti-HA, anti-H2B, and anti-β-actin) (**F**). The panels show the quantification of Nrf2 in nucleus (mean ± SD from 3 independent experiments). *** p < 0.001 as determined by unpaired two-tailed Student’s *t*-test (**G**). **H** LN229 cells treated by control, Nrf2, or p62 siRNA oligos were transfected with Flag or Flag-Smurf1. The relative mRNA of NQO1 was performed by qRT-PCR analysis. Values were normalized against the amount of mRNA in LN229 treated with control siRNA oligos and Flag; mean ± SD from 3 independent experiments. NS* p* > 0.05, * p < 0.05, ** p < 0.01, *** p < 0.001 as determined by unpaired two-tailed Student’s *t*-test. **I** LN229 cells treated by p62 siRNA oligos were transfected with Flag or Flag-Smurf1, then overexpressed GFP-p62 or GFP-p62^K7A/D69A^. Cell lysates were prepared and subjected to western blot analysis with the indicated antibodies (anti-Nrf2, anti-GFP, anti-Flag, and anti-β-actin). **J**, **K** LN229 cells treated with p62 siRNA oligos were fixed after transfecting with Flag, Flag-Smurf1, GFP-p62, or GFP-p62^K7A/D69A^, and then immunofluorescence stained with anti-Nrf2 and anti-Flag antibodies. The nucleus was stained with DAPI. Bar: 5 µm (**J**). More than 100 cells were assayed for the nuclear signal of Nrf2; mean ± SD. NS* p* > 0.05, * p < 0.05, ** p < 0.01 as determined by unpaired two-tailed Student’s *t*-test (**K**). **L** LN229 cells treated by p62 siRNA oligos were transfected with GFP-p62 or GFP-p62^K7A/D69A^, then overexpressed Flag or Flag-Smurf1. Bar graphs indicate that the relative mRNA of NQO1 was performed by qRT-PCR. Values were normalized against the amount of NQO1 mRNA in LN229 treated with si-p62, Flag, and GFP-p62; mean ± SD from 3 independent experiments. NS* p* > 0.05, *** p < 0.001 as determined by unpaired two-tailed Student’s *t*-test. **M** Role of Smurf1 in p62 droplet formation and Nrf2 activation. Smurf1 overexpression increased formation and material exchange of p62 liquid droplets. Increased p62 droplets promoted Nrf2 nuclear import

The p62-Nrf2 axis plays a critical role in protein assembly, aggregation, and degradation [23]. We aimed to verify if Smurf1 promotes the activation of Nrf2. Firstly, we observed that overexpression of Smurf1 increased, whereas knocking down Smurf1 decreased the protein expression of Nrf2 and p62 (Fig. 2A, B and Additional file 1: Fig. S2A). We also found decreased Nrf2 and p62 levels in MEFs from Smurf1 knockout (*Smurf1*^*−/−*^) mice compared to *Smurf1*^+*/*+^ MEFs (Fig. 2C). Immunofluorescence analysis showed that Smurf1 overexpression greatly enhanced the nuclear translocation of Nrf2 (Fig. 2D, E). Next, we performed a cytoplasmic and nuclear protein extraction experiment by Smurf1 overexpression (HA-Smurf1) or knockdown (si-Smurf1). Indeed, the overexpressed Smurf1 remarkably increased, and Smurf1 suppression decreased the total and nuclear portion of Nrf2 compared to the control group (Fig. 2F, G and Additional file 1: Fig. S2B). Expectedly, the expression of Nrf2 targets such as NQO1 was significantly increased by the overexpression of Smurf1 (Flag-Smurf1) compared to the control (Flag) group, while decreased by si-Smurf1 compared to non-targeting control siRNA (si-Control) group (Fig. 2H and Additional file 1: Fig. S2C). Significantly, p62 or Nrf2 knockdown reverses the overexpressed Smurf1-induced Nrf2 targets expression (Fig. 2H). To investigate if p62 phase separation plays a role in Smurf1-mediated Nrf2 activation, we overexpressed p62^K7A/D69A^ and p62^WT^ plasmid in p62-deficient LN229 cells with or without Smurf1 overexpression. We initially found that si-p62 decreases the endogenous expression of Nrf2, which was rescued by GFP-p62^WT^ overexpression but not by GFP-p62^K7A/D69A^ overexpression (Additional file 1: Fig. S2D). Importantly, overexpression of Smurf1 resulted in increased endogenous expression of Nrf2 in p62^WT^ group but not in p62^K7A/D69A^ group, suggesting Smurf1 upregulated Nrf2 in a p62 phase separation-dependent manner (Fig. 2I). We then compared the nuclear translocation percentage of Nrf2 in overexpressed GFP-p62^WT^ or GFP-p62^K7A/D69A^ cells with or without overexpressed Flag-Smurf1, and found that Smurf1 overexpression increased Nrf2 nuclear translocation in GFP-p62^WT^ overexpressed group, but not in GFP-p62^K7A/D69A^ group, compared with Flag cells, indicating that Smurf1^+^p62^+^ puncta increased Nrf2 nuclear translocation in a p62 phase separation dependent manner (Fig. 2J, K). Smurf1-induced NQO1 mRNA expression was abolished in p62^K7A/D69A^ LN229 cells (Fig. 2L). Overall, these results indicated that p62 phase separation ability is essential for Smurf1-induced activation of Nrf2 (Fig. 2M).

### Smurf1 increases the binding affinity between p62 and Keap1 to activate Nrf2

**Fig. 3:**
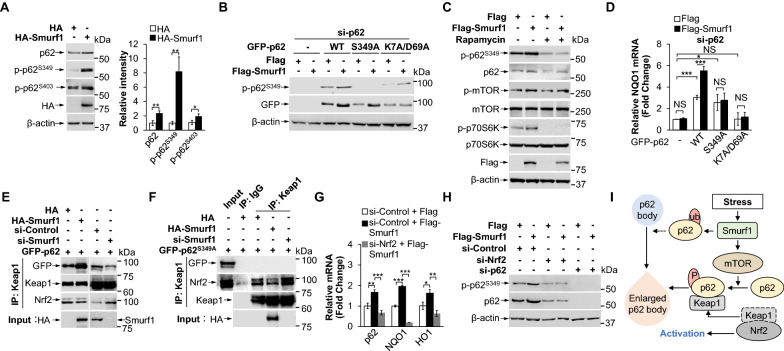
Smurf1 increases the binding affinity between p62 and Keap1 to activate Nrf2. **A** LN229 cells were transfected with HA or HA-Smurf1. Cell lysates were subjected to western blot analysis with the indicated antibodies (anti-p62, anti-p-p62^S349^, anti-p-p62^S403^, anti-HA, and anti-β-actin). Bar graphs indicate the quantitative densitometric analysis of the indicated proteins relative to β-actin (mean ± SD from 3 independent experiments). Values were normalized against the intensity of LN229 transfected with HA. * p < 0.05, ** p < 0.01 as determined by unpaired two-tailed Student’s *t*-test. **B** LN229 cells treated by p62 siRNA oligos were transfected with Flag or Flag-Smurf1, then overexpressed GFP, GFP-p62^WT^, GFP-p62^S349A^, or GFP-p62^K7A/D69A^. Cell lysates were prepared and subjected to western blot analysis with the indicated antibodies (anti-p-p62^S349^, anti-GFP, and anti-β-actin). **C** LN229 cells transfected with Flag or Flag-Smurf1 were treated with DMSO or Rapamycin (100 nM, 24 h). Cell lysates were prepared and subjected to western blot analysis with the indicated antibodies (anti-p-p62^S349^, anti-p62, anti-p-mTOR, anti-mTOR, anti-p-p70S6K, anti-p70S6K, anti-Flag and anti-β-actin). **D** LN229 cells treated by p62 siRNA oligos were transfected with Flag or Flag-Smurf1, then overexpressed GFP, GFP-p62^WT^, GFP-p62^S349A^ or GFP-p62^K7A/D69A^. Total RNAs were prepared from these LN229 cells. Bar graphs indicate the amount of NQO1 mRNA. Values were normalized against the amount of NQO1 mRNA in LN229 transfected with GFP, Flag plasmids and p62 siRNA oligos; mean ± SD from 3 independent experiments. NS* p* > 0.05, * p < 0.05, *** p < 0.001 as determined by unpaired two-tailed Student’s *t*-test. **E** 293T cells overexpressed GFP-p62 were transfected HA, HA-Smurf1, si-Control, or si-Smurf1. Cell lysates were immunoprecipitated by anti-Keap1 antibody. The immunoprecipitates and the input were probed with anti-GFP, anti-Keap1, anti-Nrf2 and anti-HA antibodies. **F** 293T cells overexpressed GFP-p62^S349A^ were transfected HA, HA-Smurf1, or si-Smurf1. Cell lysates were immunoprecipitated by anti-Keap1 antibody. The immunoprecipitates and the input were probed with anti-GFP, anti-Keap1, anti-Nrf2 and anti-HA antibodies. **G** LN229 cells treated with control or Nrf2 siRNA oligos were transfected with Flag or Flag-Smurf1. Total RNAs were prepared from these LN229 cells. Bar graphs indicate the amount of p62, NQO1, and HO1 mRNA. Values were normalized against the amount of mRNA in LN229 transfected with control siRNA oligos and Flag; mean ± SD from 3 independent experiments. * p < 0.05, ** p < 0.01, *** p < 0.001 as determined by unpaired two-tailed Student’s *t*-test. **H** LN229 cells treated with control, p62, or Nrf2 siRNA oligos were transfected with Flag or Flag-Smurf1. Cell lysates were prepared and subjected to western blot analysis with the indicated antibodies. **I** Smurf1 promotes the phosphorylation of p62^S349^ by activating mTORC1 signalling pathway. p62^S349^ phosphorylation enhance p62 binding affinity with Keap1 and Nrf2 nuclear translocation. The activated Nrf2 partially participates to the transcriptional activation of p62

Previous study shows that p62 phosphorylation at S349 increases its binding affinity with Keap1 (a negative regulator of Nrf2), thus promoting activation of Nrf2 [22]. S403 phosphorylation enhances polyubiquitin chain-induced phase separation and p62 droplet formation [5, 19]. We observed the total p62 and its phosphorylation, especially p-p62 (Ser349), were robustly increased in Smurf1 overexpressed cells and decreased in Smurf1 knockdown cells compared to the control cells, suggesting Smurf1 mediated Nrf2 activation may occur through upregulation of p62 phosphorylation at S349 (Fig. 3A and Additional file 1: Fig. S3A). To investigate whether Smurf1-induced p62 phosphorylation also depended on its phase separation ability, we overexpressed p62^S349A^, p62^K7A/D69A^ mutant, and p62^WT^ in p62-deficient LN229 cells with or without Smurf1 overexpression. We found that Flag-Smurf1 increased p-p62 (Ser349) in p62^WT^ and p62^K7A/D69A^ group but not in p62^S349A^ and control group (Fig. 3B). The p62^K7A/D69A^ hardly showed phosphorylation at S349 compared with p62^WT^, indicating that Smurf1 promoted phosphorylation S349 of p62 partly dependent of its phase separation (Fig. 3B). In addition, Flag-Smurf1 overexpression activated mTOR and increased the phosphorylation of p62 (Fig. 3C). Moreover, both phosphorylation of p62 and activation of mTOR were significantly decreased by treatment with mTOR inhibitor rapamycin, suggesting that overexpressed Smurf1 promoted the activation of mTOR to increase S349 phosphorylation of p62 (Fig. 3C). Previous studies showed that Smurf1 activated the mTORC1 signalling pathway by ubiquitination and degradation of its negative regulator PTEN (Phosphatase and Tensin Homolog deleted on Chromosome 10) [40, 41]. In order to test whether Smurf1-mediated PTEN ubiquitination is the underlying cause for mTOR activation, we knocked down Smurf1 in LN229 with si-Control or si-PTEN treatment. As expected, we found that p-mTOR was decreased by Smurf1 knockdown in control group cells, but p-mTOR was not affected by Smurf1 knockdown in PTEN defective cells (Additional file 1: Fig. S3B). In order to confirm whether PTEN is the key protein in the formation of p62 droplets, we transfected HA-PTEN in cells with Smurf1 overexpression under MG132 treatment. We found that HA-PTEN decreased Flag-Smurf1 induced p62 puncta formation (Additional file 1: Fig. S3C). Therefore, we believe that HA-PTEN partially inhibits Smurf1-mediated p62 puncta formation by inhibiting PI3K/Akt/mTOR pathway. Interestingly, Flag-Smurf1 enhanced the mRNA expression of Nrf2 target NQO1 only in p62^WT^ group, but not in p62^K7A/D69A^ and p62^S349A^ group, suggesting Smurf1 promoted Nrf2 activation in both p62^S349^ phosphorylation and its phase separation dependent manner (Fig. 3D).

Next, we tested the binding affinity of Keap1 and p62 with or without overexpressed Smurf1 under MG132 treatment. Immunofluorescent analysis revealed the number of Keap1^+^p62^+^ puncta was increased in Smurf1 overexpression group and decreased in Smurf1 or p62 knockdown group under MG132 treatment (Additional file 1: Fig. S3D-G). It also revealed the extensive co-localization of Keap1, p62, and Smurf1 in the same puncta (Additional file 1: Fig. S3E, G). Immunoprecipitation analysis found that HA-Smurf1 also increased, si-Smurf1 decreased the binding affinity between Keap1 and p62^WT^ (but not p62^S349A^) (Fig. 3E, F). Immunofluorescence assay also showed that Smurf1^+^p62^+^ puncta easily recruited Keap1 compared with p62^+^ puncta (Additional file 1: Fig. S3E).

Moreover, given that p62 is one of the targets of Nrf2, we knocked down Nrf2 in LN229 cells with or without Flag-Smurf1 overexpression to further explore the role of Nrf2 feedback. The mRNA of p62, as same as other Nrf2 target NQO1 and HO1, were increased by Flag-Smurf1 and decreased by Nrf2 knockdown (Fig. 3G). Nrf2 knockdown inhibited Flag-Smurf1 induced p62 (Fig. 3H). Overall, Smurf1 promotes the phosphorylation of p62, resulting in increased binding of p62/Keap1 and nuclear translocation of Nrf2. The activated Nrf2 partially participates in the transcriptional activation of p62 and other anti-oxidative genes (Fig. 3I).

### Smurf1 induces the autophagic degradation of p62-liquid droplets

**Fig. 4:**
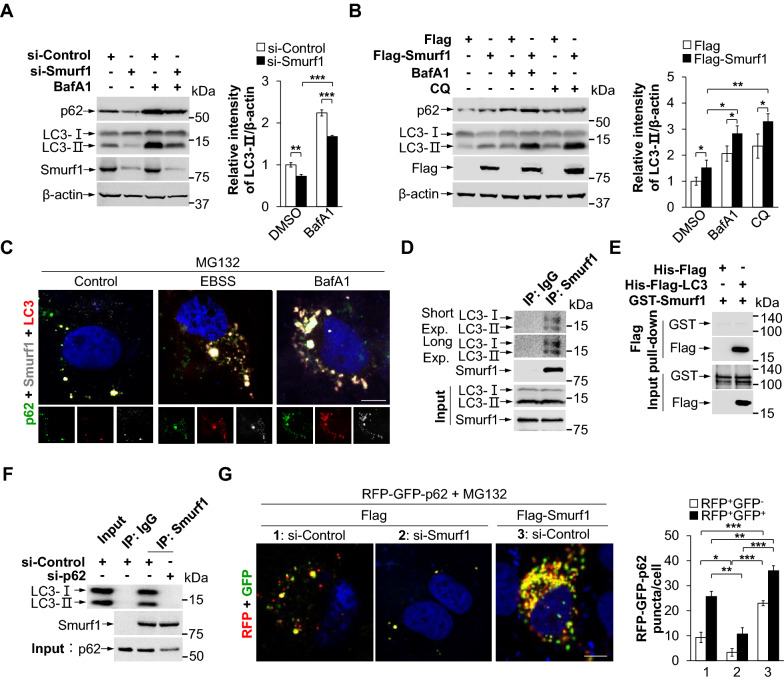
Smurf1 induces the autophagic degradation of p62-liquid droplets. **A** LN229 cells were transfected with control or Smurf1 siRNA oligos, then treated with DMSO or BafA1 (100 nM, 24 h). Cell lysates were prepared and subjected to western blot analysis with the indicated antibodies (anti-p62, anti-LC3B, anti-Smurf1, and anti-β-actin). Bar graphs indicate the quantitative densitometric analysis of LC3 relative to β-actin. Values were normalized against the intensity of LN229 transfected with si-Control and treated with DMSO; mean ± SD from 3 independent experiments. ** p < 0.01, *** p < 0.001 as determined by unpaired two-tailed Student’s *t*-test. **B** LN229 cells were transfected with Flag or Flag-Smurf1, then treated with DMSO, BafA1 (100 nM, 24 h) or CQ (100 µM, 6 h). Cell lysates were prepared and subjected to western blot analysis with the indicated antibodies (anti-Flag, anti-p62, anti-LC3B, and anti-β-actin). Bar graphs indicate the quantitative densitometric analysis of LC3 relative to β-actin. Values were normalized against the intensity of LN229 transfected with Flag and treated with DMSO; mean ± SD from 3 independent experiments. * p < 0.05, ** p < 0.01 as determined by unpaired two-tailed Student’s *t*-test. **C** LN229 cells with MG132 (20 µM, 12 h) treatment were fixed after treating with DMSO, EBSS (2 h), BafA1 (100 nM, 24 h), and then immunofluorescence stained with anti-p62, anti-Smurf1 and anti-LC3B antibodies. The nucleus was stained by DAPI. Bar: 5 µm. **D** 293T cells were treated with MG132. The lysates were immunoprecipitated by IgG or Smurf1 antibody. The immunoprecipitates and the input were probed with anti-LC3B and anti-Smurf1 antibodies. **E** The indicated His-Flag-tagged purified proteins of control and LC3 constructs were pulled down with Flag-beads separately, then incubated with GST-Smurf1. The proteins retained on beads were subjected to immunoblot using anti-GST and anti-Flag antibodies. **F** 293T cells were transfected with si-Control or si-p62 under MG132 treatment. The lysates were immunoprecipitated by IgG or Smurf1 antibody. The immunoprecipitates and the input were probed with anti-LC3B, anti-Smurf1 and anti-p62 antibodies. **G** LN229 cells treated by control or Smurf1 siRNA oligos were transfected with RFP-GFP-p62, then overexpressed Flag or Flag-Smurf1, and fixed after MG132 (20 µM, 12 h) treatment. Bar: 5 µm. The nucleus was stained by DAPI. Bar graphs indicate the number of RFP-GFP-p62 puncta in each cell (mean ± SD, n = 10 cells examined over three independent experiments). * p < 0.05, ** p < 0.01, *** p < 0.001 as determined by unpaired two-tailed Student’s *t*-test

Recent studies revealed that p62 liquid droplets were degraded by selective autophagy [20]. We wanted to investigate whether Smurf1 promotes degradation of p62 liquid droplets. Firstly, we knocked down or overexpressed Smurf1 under the treatment of autophagy inhibitor Bafilomycin (BafA1, inhibiting autophagic vesicle acidification) and Chloroquine (CQ, destroying the structure and function of lysosome). Smurf1 knockdown decreased, while overexpressed Smurf1 increased the levels of LC3-II under blockage of autophagy, suggesting Smurf1 promotes autophagosome formation (Fig. 4A, B). Next, immunofluorescent analysis revealed that autophagy inhibitor BafA1 and enhancer EBSS (starve to induce autophagy) treatment both increased autophagosome formation, evidenced by co-localized Smurf1, p62, and LC3 under proteasome stress (Fig. 4C). We immunoprecipitated the endogenous Smurf1 and found it combined with LC3 under MG132 treatment (Fig. 4D). To validate whether Smurf1 direct interact with LC3, we purified bacterially expressed GST-Smurf1 and His-Flag-LC3 proteins and conducted pull-down assays in vitro. However, no direct interaction between Smurf1 and LC3 is detected (Fig. 4E). In addition, we transfected si-Control or si-p62 in cells under MG132 treatment and found that p62 knockdown inhibited the interaction between Smurf1 and LC3, suggesting the colocation of Smurf1 and LC3 was mediated by p62 (Fig. 4F). Finally, we used RFP-GFP-p62 to detect the role of Smurf1 in the autophagic flux of p62 under the MG132 treatment. The p62 puncta possessed RFP^+^GFP^+^ signal (not in autolysosomes) and RFP^+^GFP^−^ signal (in autolysosomes) were both dramatically increased in the cells with overexpressed Smurf1 compared to the control cells, while only a small amount RFP^+^GFP^+^ signal was exhibited in the knockdown of Smurf1, further indicating that Smurf1 induces the autophagic formation and degradation of p62 (Fig. 4G).

### NBR1 enhances Smurf1-mediated p62 liquid-droplets accumulation

**Fig. 5:**
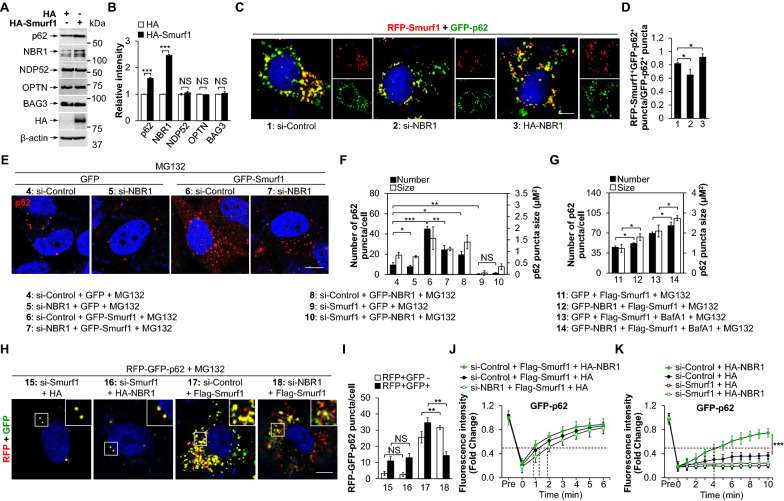
NBR1 enhances Smurf1-mediated p62 liquid-droplets accumulation. **A**, **B** LN229 cells were transfected with HA or HA-Smurf1. Cell lysates were prepared and subjected to western blot analysis with the indicated antibodies (anti-p62, anti-NBR1, anti-NDP52, anti-OPTN, anti-BAG3, anti-HA, and anti-β-actin) (**A**). Bar graphs indicate the quantitative densitometric analysis of the indicated proteins relative to β-actin. Values were normalized against the intensity of LN229 transfected with HA; mean ± SD from 3 independent experiments. NS* p* > 0.05, *** p < 0.001 as determined by unpaired two-tailed Student’s *t*-test (**B**). **C**, **D** LN229 cells transfected with RFP-Smurf1 and GFP-p62 were treated with control or NBR1 siRNA oligos, fixed after 24 h transfection with HA or HA-NBR1. The nucleus was stained by DAPI. Bar: 5 µm (**C**). Bar graphs indicate the ratio of the number of RFP-Smurf^+^GFP-p62^+^ puncta to the number of GFP-p62^+^ puncta in each cell (mean ± SD, n = 10 cells examined over three independent experiments). * p < 0.05 as determined by unpaired two-tailed Student’s *t*-test (**D**). **E**, **F** LN229 cells treated with control, NBR1, or Smurf1 siRNA oligos were transfected with GFP, GFP-Smurf1, or GFP-NBR1, fixed after treating with MG132 (20 µM, 12 h), and then immunofluorescence stained with anti-p62 antibody. Bar: 5 µm. The nucleus was stained by DAPI (**E**). Bar graphs indicate the number and size of p62 puncta in each cell (mean ± SD, n = 10 cells examined over three independent experiments). NS* p* > 0.05, * p < 0.05, ** p < 0.01, *** p < 0.001 as determined by unpaired two-tailed Student’s *t*-test (**F**). **G** LN229 cells overexpressing Flag-Smurf1 were transfected with GFP or GFP-NBR1, fixed after treating with BafA1 (100 nM, 24 h) and/or MG132 (20 µM, 12 h), and then immunofluorescence stained with anti-p62 antibody. Bar graphs indicate the number and size of p62 puncta in each cell (mean ± SD, n = 10 cells examined over three independent experiments). NS* p* > 0.05, * p < 0.05 as determined by unpaired two-tailed Student’s *t*-test. **H**, **I** LN229 cells transfected with RFP-GFP-p62 were treated by control, Smurf1 or NBR1 siRNA oligos, treated with MG132 (20 µM, 12 h), and fixed after transfection with HA, HA-NBR1 or Flag-Smurf1. The nucleus was stained by DAPI. Bar: 5 µm (**H**). Bar graphs indicate the number of RFP-GFP-p62 puncta in each cell (mean ± SD, n = 10 cells examined over three independent experiments). NS* p* > 0.05, ** p < 0.01 as determined by unpaired two-tailed Student’s *t*-test (**I**). **J** LN229 cells transfected with GFP-p62 and Flag-Smurf1 cultured in glass-bottom plates were treated with control or NBR1 siRNA oligos, then overexpressed HA or HA-NBR1. The signal recovery after photobleaching was measured; mean ± SD, n = 20 droplets examined over three independent experiments. **K** LN229 cells transfected with GFP-p62 were treated with control or Smurf1 siRNA oligos, and overexpressed HA or HA-NBR1. The signal recovery after photobleaching was measured; mean ± SD, n = 20 droplets examined over three independent experiments

The p62 protein has been shown to act cooperatively with autophagy receptors NBR1, OPTN, and NDP52 [24, 30, 31]. To further study the mechanism of Smurf1 mediated p62 liquid-droplets formation, we first identified that the overexpression of Smurf1 increased, while Smurf1-depleted specifically decreased p62 and NBR1, but not other autophagy receptors like OPTN, NDP52, and BAG3 (Fig. 5A, B and Additional file 1: Fig. S4A). Immunofluorescence staining also showed overexpression of Smurf1 increased, whereas knocking down Smurf1 decreased the co-localization of p62 and NBR1 (Additional file 1: Fig. S4B). NBR1 shares a remarkable structural similarity with p62 and induced p62 liquid droplet formation [18, 32]. To confirm the promoting effect of NBR1 in Smurf1-mediated p62 liquid-droplets formation, we transfected si-Control, si-NBR1, or HA-NBR1 in RFP-Smurf1 and GFP-p62 overexpressed LN229 cells. We found that NBR1 knockdown decreased, and NBR1 overexpression increased the number of RFP-Smurf1^+^GFP-p62^+^ puncta, suggesting that NBR1 promoted the Smurf1-mediated p62 formation (Fig. 5C, D). However, GFP-NBR1-activated effect was diminished under Smurf1 knockdown, indicating that Smurf1, but not NBR1, is necessary for p62 droplet formation (Fig. 5E, F).

To further verify the role of NBR1 in Smurf1-mediated autophagic degradation of p62 droplets, we overexpressed NBR1 in Flag or Flag-Smurf1 LN229 under BafA1 treatment. We found that overexpression of NBR1 slightly increased the number of p62 puncta in Flag-Smurf1 overexpressed LN229 under the treatment of BafA1 (Fig. 5G). We also used RFP-GFP-p62 to indicate autophagy flux. Significantly, NBR1 knockdown showed decreased number of RFP^+^GFP^+^ signals (not in autolysosomes) and increased number of RFP^+^GFP^−^ sign (in autolysosomes) compared with si-Control in Flag-Smurf1 LN229 cells (Fig. 5H, I). These data showed that NBR1 promotes Smurf1-mediated p62 puncta accumulation by promoting p62 droplets formation and blocking autophagic degradation.

To further verify whether NBR1 promotes Smurf1-mediated p62-phase separation, we tested the exchange of components by the FRAP assay. We found that overexpression NBR1 slightly increased (1 ± 0.5 min), and NBR1 knockdown (2 ± 1 min) decreased the* t*1/2 of p62 liquid droplets compared to control in Smurf1 overexpressed LN229 cells, suggesting NBR1 promoted the liquid properties of Smurf1-induced p62 puncta (Fig. 5J). We overexpressed NBR1 in cells with si-Control or si-Smurf1 and found that NBR1 overexpression significantly shortened the* t*1/2 of p62 liquid droplets (5 ± 2 min) in si-Control group compared to si-Smurf1 group in LN229 (Fig. 5K). Interestingly, the* t*1/2 of p62 signal intensity also has no statistical difference between NBR1 knockdown and control in si-Smurf1 LN229 cells, suggesting NBR1 is not required for the Smurf1-induced liquid properties of p62 droplet (Additional file 1: Fig. S4C). Collectively, our data indicate that NBR1 promotes but is not necessary for Smurf1-mediated p62 liquid-droplets formation and autophagy flux.

### Smurf1 mediated NBR1 expression in p62 dependent manner

**Fig. 6:**
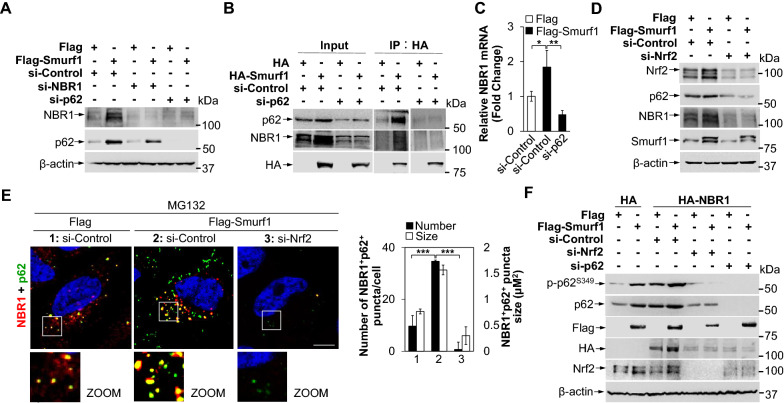
Smurf1 mediated NBR1 expression in p62 dependent manner. **A** LN229 cells treated with control, p62 or NBR1 siRNA oligos were transfected with Flag or Flag-Smurf1. Cell lysates were prepared and subjected to western blot analysis with the indicated antibodies (anti-NBR1, anti-p62, and anti-β-actin). **B** 293T cells treated with control or p62 siRNA oligos were transfected with HA or HA-Smurf1, and immunoprecipitated by HA antibody. The immunoprecipitates and the input were probed with anti-p62, anti-NBR1, and anti-HA antibodies. **C** LN229 cells treated with control or p62 siRNA oligos were transfected with Flag or Flag-Smurf1. Total RNAs were prepared from these LN229 cells. Bar graphs indicate the amount of NBR1 mRNA. Values were normalized against the amount of LN229 transfected with si-Control and Flag; mean ± SD from 3 independent experiments. * p < 0.05, ** p < 0.01 as determined by unpaired two-tailed Student’s *t*-test. **D** LN229 cells treated with control or Nrf2 siRNA oligos were transfected with Flag or Flag-Smurf1. Cell lysates were prepared and subjected to western blot analysis with the indicated antibodies (anti-Nrf2, anti-p62, anti-NBR1, anti-Smurf1 and anti-β-actin). **E** LN229 cells treated with control or Nrf2 siRNA oligos were transfected with Flag or Flag-Smurf1, fixed after treating with MG132 (20 µM, 12 h), and then immunofluorescence stained with anti-NBR1 and anti-p62 antibodies. The nucleus was stained by DAPI. Bar: 5 µm. Bar graphs indicate the number and size of NBR1^+^p62^+^ puncta in each cell (mean ± SD, n = 10 cells examined over three independent experiments). *** p < 0.001 as determined by unpaired two-tailed Student’s *t*-test. **F** LN229 cells treated with control, Nrf2, or p62 siRNA oligos were transfected with HA or HA-NBR1, and then overexpressed Flag or Flag-Smurf1. Cell lysates were prepared and subjected to western blot analysis with the indicated antibodies (anti-p-p62^S349^, anti-p62, anti-Flag, anti-HA, anti-Nrf2, and anti-β-actin)

Next, we knocked down NBR1 or p62 in LN229 cells with Smurf1 overexpression or Smurf1 knockdown. We found that the Smurf1-mediated increase of NBR1 depended on p62 protein level, evidenced by the p62 knockdown diminished the response of NBR1 to overexpression or knockdown of Smurf1 (Fig. 6A and Additional file 1: Fig. S5A). To determine whether p62 induced the interaction between NBR1 and Smurf1, we transfected si-p62 or si-Control in HA-Smurf1 or HA LN229 cells. We detected NBR1 and p62 along with the immunoprecipitation of HA-Smurf1. Notably, the interaction of Smurf1 and NBR1 disappeared in the absent p62, suggesting p62 plays a bridge role between Smurf1 and NBR1 (Fig. 6B).

In addition, we found that Smurf1 overexpression leads to increased NBR1 mRNA levels (Fig. 6C). To test whether the increased NBR1 transcriptional levels are mediated by Smurf1-induced activation of Nrf2, we knocked down Nrf2 in Flag-Smurf1 LN229 cells and overexpressed Nrf2 in si-Smurf1 LN229 cells. Nrf2 knockdown decreased both NBR1 protein level and co-localization of p62 and NBR1 in Flag-Smurf1 cells compared with si-Control (Fig. 6D, E). Myc-Nrf2 increased both NBR1 protein level and co-localization of p62 and NBR1 in LN229 cells transfected si-Smurf1 compared with Myc control (Additional file 1: Fig. S5B, C). Nrf2 or p62 knockdown reduces HA-NBR1 protein levels compared to si-Control, suggesting that Nrf2 is a mediator to increase Smurf1-induced NBR1 expression (Fig. 6F). We also found that overexpression of NBR1 further increased protein level and phosphorylation level of p62 under Smurf1 overexpression (Fig. 6F). Of note, NBR1 knockdown decreased, and NBR1 overexpression increased Smurf1-induced mRNA level of NQO1 compared with control LN229 cells (Additional file 1: Fig. S5D). Defective p62 phase separation reduced mRNA levels of NQO1 in LN229 with Flag-Smurf1 and HA-NBR1, suggesting that p62 phase separation is necessary for NBR1-induced Nrf2 activation (Additional file 1: Fig. S5E).

### NBR1 enhances Smurf1-drived Nrf2-mediated oxidative stress response

**Fig. 7:**
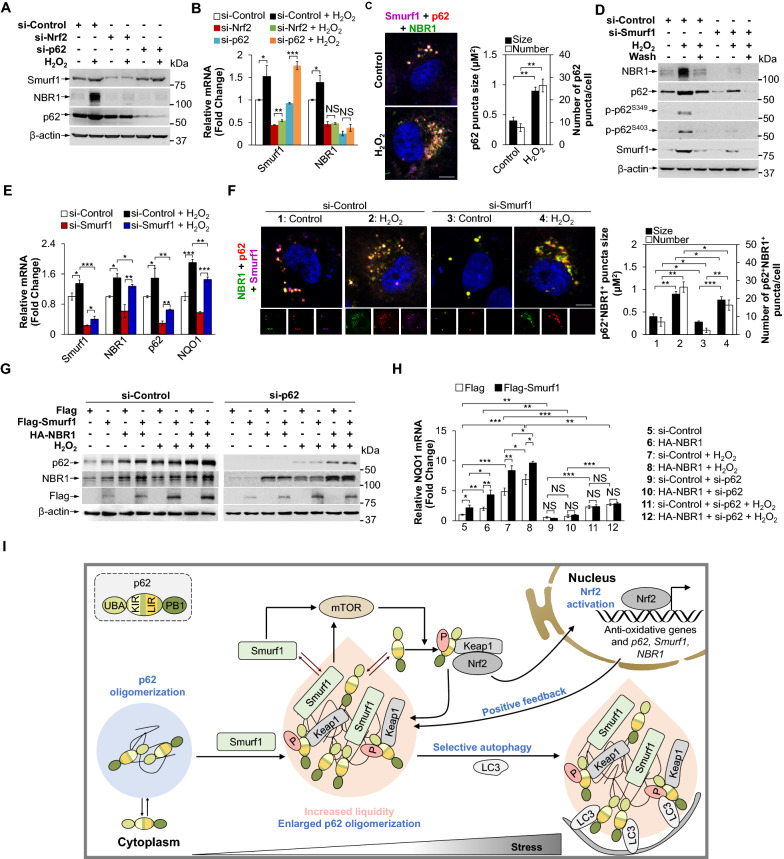
NBR1 enhances Smurf1-drived Nrf2-mediated oxidative stress response. **A** LN229 cells transfected with control, Nrf2 or p62 siRNA oligos were treated with control (PBS) or H_2_O_2_ (200 µM, 2 h). Cell lysates were prepared and subjected to western blot analysis with the indicated antibodies (anti-Smurf1, anti-NBR1, anti-p62, and anti-β-actin). **B** LN229 cells transfected with control, Nrf2 or p62 siRNA oligos were treated with control (PBS) or H_2_O_2_ (200 µM, 2 h). Total RNAs were prepared from these LN229 cells. Bar graphs indicate the amount of Smurf1 and NBR1 mRNA. Values were normalized against the amount of mRNA in LN229 treated with control siRNA oligos and control (PBS) (mean ± SD from 3 independent experiments). NS* p* > 0.05, * p < 0.05, ** p < 0.01, *** p < 0.001 as determined by unpaired two-tailed Student’s *t*-test. **C** LN229 cells were fixed after treating with control (PBS) or H_2_O_2_ (200 µM, 2 h), and then immunofluorescence stained with anti-Smurf1, anti-p62, and anti-NBR1 antibodies. The nucleus was stained by DAPI. Bar: 5 µm. Bar graphs indicate the number and size of p62 puncta in each cell (mean ± SD, n = 10 cells examined over three independent experiments). ** p < 0.01 as determined by unpaired two-tailed Student’s *t*-test. **D** LN229 cells were transfected with control or Smurf1 siRNA oligos. Thereafter, the cells were divided into three groups: (i) cultured in regular medium, (ii) treated with H_2_O_2_ (200 µM, 2 h), and (iii) after treating with H_2_O_2_ (200 µM, 2 h), cultured in regular medium for another 12 h. Cell lysates were prepared and subjected to western blot analysis with indicated antibodies (anti-NBR1, anti-p62, anti-p-p62^S349^, anti-p-p62^S403^, anti-Smurf1, and anti-β-actin). **E** LN229 cells transfected with control or Smurf1 siRNA oligos were treated with control (PBS) or H_2_O_2_ (200 µM, 2 h). Total RNAs were prepared from these LN229 cells. Bar graphs indicate the amount of Smurf1, p62, NBR1, and NQO1 mRNA. Values were normalized against the amount of mRNA in LN229 treated with control siRNA oligos and control (PBS) (mean ± SD from 3 independent experiments). NS* p* > 0.05, * p < 0.05, ** p < 0.01, *** p < 0.001 as determined by unpaired two-tailed Student’s *t*-test. **F** LN229 cells treated with control or Smurf1 siRNA oligos and fixed after treating with control (PBS) or H_2_O_2_ (200 µM, 2 h), and then immunofluorescence stained with anti-NBR1, anti-Smurf1 and anti-p62 antibodies. The nucleus was stained by DAPI. Bar: 5 µm. Bar graphs indicate the number and size of p62 puncta in each cell (mean ± SD, n = 10 cells examined over three independent experiments). * p < 0.05, ** p < 0.01, *** p < 0.001 as determined by unpaired two-tailed Student’s *t*-test. **G** LN229 cells transfected with control or p62 siRNA oligos were transfected with Flag or Flag-Smurf1, and treated with control (PBS) or H_2_O_2_ (200 µM, 2 h) after overexpressing HA or HA-NBR1. Cell lysates were prepared and subjected to western blot analysis with the indicated antibodies (anti-p62, anti-NBR1, anti-Flag, and anti-β-actin). **H** LN229 cells transfected with control or p62 siRNA oligos were transfected with Flag or Flag-Smurf1, and treated with control (PBS) or H_2_O_2_ (200 µM, 2 h) after overexpressing HA or HA-NBR1. Total RNAs were prepared from these LN229 cells. Bar graphs indicate the amount of NQO1 mRNA. Values were normalized against the amount of mRNA in LN229 transfected with control siRNA oligos and Flag; mean ± SD from 3 independent experiments. NS* p* > 0.05, * p < 0.05, ** p < 0.01, *** p < 0.001 as determined by unpaired two-tailed Student’s *t*-test. **I** Role of Smurf1 in stress response. Stress upregulates the level of Smurf1, leading to the increased formation and material exchange of p62 liquid droplets. Smurf1 promotes the phosphorylation of p62^S349^ by activating mTORC1 signalling pathway. These further promote the transcription of anti-stress proteins by competitively binding Keap1 and mediating Nrf2 nuclear import. The activated Nrf2 increases the mRNA level of Smurf1, p62, and NBR1 to promote the formation and enlarging of p62 liquid droplets as positive feedback

Given that p62 liquid droplet reduces reactive oxygen species (ROS) production by recruiting Keap1 and subsequently activating the transcription factor Nrf2 [21, 33, 34], we sought to test if Smurf1 plays a role in oxidative stress response. Initially, to investigate the expression of endogenous Smurf1 upon exposure to oxidative stress, we transfected LN229 cells with si-Nrf2 or si-p62 under H_2_O_2_ treatment. The results showed that H_2_O_2_ dramatically increased the expression of Smurf1, NBR1, and p62 in Nrf2 dependent manner (Fig. 7A). The qRT-PCR analysis also revealed that the mRNA levels of Smurf1 and NBR1 significantly decreased under Nrf2 knockdown (Fig. 7B). These results indicated that Nrf2 is a transcription factor for Smurf1 and NBR1 in response to oxidative response. We found that Smurf1-p62-NBR1 co-localized in LN229 with or without H_2_O_2_ treatment, and H_2_O_2_ treatment increased the number and size of p62^+^NBR1^+^ liquid droplets in agreement with Smurf1 overexpression (Fig. 7C). To further confirm whether stress-induced Smurf1 expression contributes to p62-mediated Nrf2 activation, we knocked down Smurf1 in LN229 cells with H_2_O_2_ treatment. We observed the H_2_O_2_ accumulated NBR1, Smurf1, total and phosphorylated p62 in the presence of Smurf1, compared with Smurf1-deficient LN229 (Fig. 7D). The qRT-PCR analysis revealed that H_2_O_2_ increased the mRNA level of NBR1, p62, and NQO1 (Fig. 7E). Smurf1 suppression reduces the mRNA levels of NBR1, p62, and NQO1 upon exposure to H_2_O_2_, indicating that Smurf1 activates Nrf2 in response to oxidative stress (Fig. 7E). Furthermore, we found that the deletion of Smurf1 significantly reduces the number and size of p62^+^NBR1^+^ liquid droplets in LN229 with or without H_2_O_2_ treatment (Fig. 7F).

Consistently, NBR1 overexpression increased H_2_O_2_ and Flag-Smurf1-induced p62 (Fig. 7G). NBR1 knockdown also slightly reduced the protein level of p62 in cells with H_2_O_2_ treatment (Additional file 1: Fig. S6A). NBR1 overexpression increased the mRNA level of NQO1 in cells with or without H_2_O_2_ treatment (Fig. 7H). NBR1 knockdown also reduced the mRNA level of NQO1 in cells with H_2_O_2_ treatment, indicating that NBR1 inhibition reduced the oxidative stress response (Additional file 1: Fig. S6B). Additionally, p62 knockdown decreased H_2_O_2_ and Flag-Smurf1-induced accumulation of NBR1 (Fig. 7G and Additional file 1: Fig. S6A). Similarly, our results showed that the p62 knockdown reduced the mRNA level of NQO1 in cells with or without H_2_O_2_ treatment (Fig. 7H and Additional file 1: Fig. S6B). Cotreatment of NBR1 and p62 knockdown could not further reduce the mRNA level of NQO1 compared with p62 knockdown alone, which confirmed that the corresponding promoting effect of NBR1 on oxidative stress depended on p62 (Additional file 1: Fig. S6B).

Collectively, these data suggest that Smurf1 positively modulates the formation of p62 puncta. Taken together, our results indicate that Smurf1 is oxidative stress (H_2_O_2_)-inducible protein. It collaborated with p62 and NBR1 during the activation of the Keap1-Nrf2 oxidative stress response pathway, promoting the formation and degradation of p62-liquid droplets and Nrf2 full-activation (Fig. 7I).

## Discussion

Stress-induced scaffold protein p62 helps cells cope with oxidative stress by activating the Nrf2 pathways. It also serves as a selective autophagy receptor that drives ubiquitinated cargos toward autophagic degradation [20–22]. A previous report showed that p62 could cause LLPS for liquid droplet formation [18]. However, the regulatory linkage between p62 liquid droplet formation and activation of Nrf2 is still missing. In this study, we identified Smurf1 as a key regulatory protein to induce a comprehensive response to proteotoxic or oxidative stresses by facilitating the biogenesis of p62 liquid droplets and degradation of ubiquitinated cargos (Fig. 7I). Mechanistically, Smurf1 promotes the phase separation of p62 to increase Nrf2 nuclear translocation in response to oxidative stress. Nrf2 activation further increases the mRNA levels of Smurf1, p62, and NBR1 as a positive feedback loop.

Studies have shown that the formation of p62 liquid droplets is promoted by either increasing the p62 protein level and/or enhancing the interaction of p62 with the ubiquitinated substrates [5, 18, 19]. For example, the transcription factors (Nrf2, nuclear factor kappa-B (NF-κB), and MiTF/TFE (microphthalmia/transcription factor E)) upregulated the p62 expression in response to various stresses [35–37]. The Keap1-Nrf2 system is involved in the cytoprotective response to oxidative stress and other cellular stresses. Keap1 constitutively suppresses Nrf2 activity, and many factors disrupt the Keap1-Nrf2 interaction. E3 ligase TRIM25  (Tripartite motif-containing 25) directly targets Keap1 by ubiquitination and degradation, leading to Nrf2 activation [38]. TRIM16 (Tripartite motif-containing 16) promoted K63-linked ubiquitination of Nrf2 to promote its stability and activation [23]. In this study, we identified that Smurf1 promoted the activation of Nrf2 by preventing the Keap1-mediated proteolysis through LLPS mechanism. The FRAP assay showed that Smurf1 interacted with p62 and increase the fluorescence recovery of p62 (Fig. 1E, K). The loss of Smurf1 reduced the size and the number of p62 droplets and decreased the recovery of signal intensity of structures positive for GFP-p62 after photobleaching (Fig. 1C, D) and Fig. 5K). Smurf1 promotes the formation of p62 liquid droplets via the following two mechanisms. To begin with, mTORC1 signalling plays a well-established role in regulating nutrient metabolism and autophagy [39]. Previous studies showed that Smurf1 activated the mTORC1 signalling pathway by ubiquitination and degradation of its negative regulator PTEN [40, 41]. The mTORC1 acutely controlled the autophagic flux through either direct phosphorylation of proteins required for the initiation of autophagosome formation, such as ULK1 (unc-51-like autophagy-activating kinase 1) and Atg13 (autophagy-related proteins 13), or indirect phosphorylation (Ser349) of p62 [42, 43]. Also, mTORC1 involved p62^S349^ phosphorylation in enhancing p62 binding affinity with Keap1 [22]. S349 is an essential regulatory phosphorylation site in p62 as it is in the KIR (Keap1 interacting region) motif interacting with the Nrf2 inhibitor Keap1 [22]. The phosphorylation of this site increases affinity for Keap1, sequestrating into p62 liquid droplets. The overexpression of Smurf1 accompanied the robust phosphorylation of p62 at S349 to activate Nrf2 (Fig. 3). Our results further identified that Smurf1 promoted p62^S349^ phosphorylation in mTORC1-dependent manner, which might increase the Keap1 fusion with the p62 liquid droplets (Fig. 3). PTEN overexpression partially decreased Smurf1 activation-mediated p62 puncta formation by inhibiting PI3K/Akt/mTOR pathway (Additional file 1: Fig. S3C), indicating that PTEN may not completely sufficient to block Smurf1 mediated p62 droplets formation. Because Smurf1 interact with p62 and promote the ubiquitination of p62, whether and how Smurf1 directly regulates p62 to enhance the formation of p62 droplets is worth further exploration. Furthermore, the UBA (ubiquitin-association) domain of p62 phosphorylation at S403 by casein kinase 2 (CK2), TANK-binding kinase 1 (TBK1), or ULK1 enhanced the binding affinity of p62 to ubiquitin chains for autophagic clearance of ubiquitinated proteins in a phase separation-dependent manner [5, 19, 44–46]. We also found that the overexpression of Smurf1 also accompanied the increased phosphorylation of p62 at S403 (Fig. 3A). However, it is still worthwhile to study the direct kinase and the role of p62 S403 phosphorylation mediated by the overexpression of Smurf1. In addition, Keap1/CUL3 ubiquitinates p62 on the UBA domain to increase the formation of ubiquitinated inclusion bodies and the size of p62 droplets [47]. SPOP interacts with p62 through p62-SBC motif and SPOP-MATH domain to promote the p62-UBA domain/ubiquitin combination and the p62 phase separation [48]. Whether Smurf1 affects droplet formation through UBA domain of ubiquitinated p62 deserves further exploration.

Functionally, Smurf1-induced p62 liquid droplets facilitate the activation of Nrf2 and autophagic degradation to maintain cellular homeostasis. On the one hand, Smurf1 promotes p62 phase separation to activate the Keap1-Nrf2 antioxidant pathway. p62 liquid droplets recruit Keap1 to prevent Keap1-mediated Nrf2 proteolysis, allowing Nrf2 translocation into the nucleus to induce the downstream target genes of antioxidant enzymes [23, 49]. Our study suggested that Smurf1-induced p62 phase condensation was critical for Keap1 recruitment, promoting Nrf2 activation and stress response (Fig. 3). Smurf1 suppression reduced the size and number of p62 droplets and decreased Nrf2 activation induced by oxidative stress (Fig. 7E, F). This induced Nrf2-mediated transcription of oxidative responsive genes (*p62, NQO1*), thereby increasing p62 liquid droplet formation. Most importantly, Smurf1 is also upregulated by Nrf2 in oxidative stress (Fig. 7B). Under oxidative stress, Nrf2 activation increases the protein levels of Smurf1, p62, and NBR1, which further promotes the phase separation of p62 (Fig. 7A). On the other hand, p62 provides a molecular link between simultaneously binding polyubiquitinated proteins and LC3 conjugated to the phagophore membrane [21, 50]. S349 phosphorylation is involved in the interaction of p62 with FIP200 to initiate selective autophagy and transport droplets into the lysosome [20, 51]. Whether Smurf1 activates selective autophagy in a p62 S349 phosphorylation-dependent manner needs further exploration.

Moreover, we found that Smurf1 increased the protein level of NBR1 and further promoted p62 liquid droplet formation. NBR1 also promotes p62 phase separation to enhance the Nrf2 system [18]. However, the promoting effects of Smurf1 and NBR1 on p62 droplets are different. NBR1 was unnecessary for p62 phase separation, evidenced by the fact that NBR1 overexpression did not increase p62 liquid droplet formation in Smurf1 knockdown cells. In addition, NBR1 overexpression inhibits the degradation of Smurf1-mediated p62 droplets in cells (Fig. 5H, I). Surprisingly, we found that the fluorescence of many p62^+^ puncta could not be restored after bleaching in NBR1-knockdown LN229 cells. We speculated that NBR1 knockdown might limit the number of p62-positive puncta in autophagosomes by membrane encapsulation. Different from NBR1, Smurf1 is required for p62 phase separation under conditions of proteotoxic and oxidative stresses. Smurf1 knockdown reduces the number and size of p62 droplets and inhibits the exchange of p62 droplets with the outside world, indicating that Smurf1 plays a key role in phase separation (Fig. 5K). Smurf1 knockdown inhibited Nrf2 activation and decreased the p62 protein level, suggesting that Smurf1 was responsible for p62 transcription under stress (Additional file 1: Fig. S2A, C).

In conclusion, we showed that Smurf1 induced the formation and material exchange of p62 liquid droplets to promote autophagosome degradation. Smurf1 promoted the formation of p62 liquid droplets to activate Nrf2 in response to proteotoxic or oxidative stress. We found that Smurf1 also increased the protein level of NBR1 to promote p62 liquid droplet formation and fully activated Nrf2 response to oxidative stress. Activated Nrf2 increased p62, Smurf1, and NBR1 transcription and further promoted the formation of p62 liquid droplets. This study provided a novel mechanism for Smurf1 to participate in p62-dependent selective autophagy through the LLPS mechanism. It also implied that overexpressed Smurf1 in tumor cells protected them from stress damage by activating Nrf2 signal pathways.

## Methods

### Cell culture and transfections

Human embryonic kidney 293T (HEK293T) and human glioblastoma cell lines LN229 were purchased from the American Type Culture Collection (ATCC) and reauthenticated by Short Tandem Repeat (General Biosystems (Anhui) Corporation Limited). Cells were grown in DMEM (Gibco, C11995500BT) media supplemented with 10% fetal bovine serum (FBS; Hyclone), and penicillin-streptomycin (100 U/mL penicillin, 100 µg/mL streptomycin, Thermo Fisher Scientific, 15140163), a humidified atmosphere of 5% CO_2_ at 37 °C.

Cells were transfected with plasmids or siRNA using Lipofectamine 2000 reagent (Invitrogen) or Lipofectamine® RNAiMax Reagent (Invitrogen) following the supplier’s instructions. The cells were collected and analyzed by western blot.

Cells were treated with dimethyl sulfoxide (DMSO, Solarbio, D8371), Z-Leu-Leu-Leu-al (MG132, MCE, HY-13259), EBSS (Gibco,14155-063), Bafilomycin (MCE, HY-100558), Rapamycin (Selleckchem, AY-22989) or H_2_O_2_ (Beijing Tong Guang Fine Chemicals Company, 7722-84-1).

The following human siRNAs were used: si-Control was purchased from JTS scientific; si-Smurf1: 5ʹ-GCGUUUGGAUCUAUGCAAATT-3ʹ; si-p62: 5ʹ-GGCUGAAGGAAGCUGCCUU-3ʹ; si-Nrf2: 5ʹ-GAAUGGUCCUAAAACACCATT-3ʹ; si-NBR1: 5ʹ-AAACCUGACUUUGGCUUCCACAGAA-3ʹ.

### Quantitative RT-PCR

Total RNA was extracted from each sample using TRNzol Universal reagent (Tiangen, DP424) according to the manufacturer’s instructions. 500 ng of RNA was used as a template to generate cDNA using the ABScript II RT Mix for qRT-PCR with gDNA Remover (ABclonal, RK20403) according to the manufacturer’s instructions. For PCR amplification of cDNA, 5 μl of the reverse transcription reaction solution was used with sense and anti-sense primers of the gene of interest using 2 × Universal SYBR Green Fast qRT-PCR Mix (ABclonal, RK21203), and primers corresponding to β-actin were included as an internal control. Primer sequences were NBR1, 5ʹ-AGGAGCAAAACGACTAGCTGC-3ʹ(forward) and 5ʹ-TCTGGGGTCTTCATGTCTGAT-3ʹ(reverse); p62, 5ʹ-GACTACGACTTGTGTAGCGTC-3ʹ(forward) and 5ʹ-AGTGTCCGTGTTTCACCTTCC-3’(reverse); β-actin, 5ʹ-CATGTACGTTGCTATCCAGGC-3ʹ(forward) and 5ʹ-CTCCTTAATGTCACGCACGAT-3ʹ(reverse); Smurf1, 5ʹ-AGATCCGTCTGACAGTGTTATGT-3’(forward) and 5ʹ-CCCATCCACGACAATCTTTGC-3ʹ(reverse); NQO1, 5ʹ-GAAGAGCACTGATCGTACTGGC-3ʹ(forward) and 5ʹ-GGATACTGAAAGTTCGCAGGG-3’(reverse); HO1, 5ʹ-AAGACTGCGTTCCTGCTCAAC-3ʹ(forward) and 5ʹ-AAAGCCCTACAGCAACTGTCG-3ʹ(reverse).

### Western blot

Western blot was used to analyze protein expression as described. In brief, cells were collected and lysed with RIPA buffer (50 mM Tris-HCl, pH 8.0; 150 mM NaCl; 1% NP40; 0.5% sodium deoxycholate; 0.1% SDS) supplemented with 1 mM PMSF and 1% phosphatase inhibitor (BOSTER). Protein lysates with 5 × loading buffer (60 mM Tris-HCl, pH 6.8; 2% SDS; 0.1% Bromophenol blue; 25% Glycerol; 14.4 mM β-Mercaptoethanol) were sonicated and boiled for 10 min. Protein samples were first resolved by SDS-PAGE, then transferred to a nitrocellulose membrane, and subsequently incubated with the primary antibody. After incubation with peroxidase-conjugated secondary antibodies, the signals were visualized by enhanced chemiluminescence according to the manufacturer’s instructions. The following primary antibodies were used: p62 (ENZO, BML-PW9860; MBL, PM045; MBL, M162-3), p-p62 Ser403 (Cell Signaling Technology, #39786), p-p62 Ser349 (Cell Signaling Technology, #16177), β-actin (Sigma, A1978), Nrf2 (Proteintech, 16396-1-AP), NDP52 (Proteintech, 12229-1-AP), NBR1 (Proteintech, 160004-1-AP), LC3B (Sigma, L7543), OPTN (Santa, sc-271549), Smurf1 (Santa, sc100616; Abcam, ab57573), GFP (Proteintech, 66002-1-lg), HA (MBL, M180-3), Flag (Sigma, F1804), c-Myc (Proteintech, 16286-1-AP), Keap1 (Proteintech, 10503-2-AP), BAG3 (Santa, sc-136467), p-mTOR (Ser2448) (Cell Signaling Technology, #2971), mTOR (Cell Signaling Technology, #2983), p-p70S6K (Thr389) (Cell Signaling Technology, #9205), p70S6K (Santa, sc-8418). The following secondary antibodies were used: Horseradish Peroxidase-conjugated goat anti-rabbit IgG (BOSTER, BA1054), Horseradish Peroxidase-conjugated goat anti-mouse IgG (BOSTER, BA1050).

### Cytoplasmic and nuclear protein extraction

Cells were collected and lysed with sucrose buffer (320 mM Sucrose; 3 mM CaCl_2_; 2 mM MgAc; 0.1 mM EDTA; 1 mM DTT; 0.5 mM PMSF) with 0.5% NP40 for 40 min on ice. The sample was fractionated after centrifugation at 600 × g for 15 min at 4℃. The supernatant was collected as cytoplasmic fraction. The precipitate was washed using sucrose buffer and centrifugated at 600 × g for 10 min at 4℃ three times. The precipitate was resuspended using RIPA buffer (50 mM Tris-HCl, pH 8.0; 150 mM NaCl; 1% NP40; 0.5% sodium deoxycholate; 0.1% SDS) supplemented with 1 mM PMSF and 1% phosphatase inhibitor (BOSTER), and used as nuclear fraction after sonication.

### Immunoprecipitation

Cells were lysed with lysis buffer (50 mM Tris, pH8.0; 150 mM NaCl; 1% NP40; 0.5% sodium deoxycholate) supplemented with 1 mM PMSF and 1% phosphatase inhibitor (BOSTER), and then sonicated with bath sonicator, centrifuged at 12,000 rpm for 10 min at 4 ℃. The supernatant of lysates was incubated with primary antibody and protein G agarose beads (Solarbio, R8300) at 4 ℃. The immunocomplexes were washed with ice-cold 1 × PBS four times and then boiled in the SDS sample buffer for 10 min. Both lysates and immunoprecipitates were identified by western blot.

### Fluorescence recovery after photobleaching (FRAP)

Cells were grown in glass base dishes. FRAP assay was conducted using the FRAP module of the NIKON N-SIM E confocal microscopy system. Proteins with fluorescent label GFP and RFP were bleached using a 488 nm and a 555 nm laser beam separately. Bleaching was focused on a circular region of interest (ROI) using 70% laser power (3 s) and time-lapse images were collected. Fluorescence intensity was measured using Image J software.

### Immunofluorescence microscopy

Cells were cultured on coverslips, fixed in 4% paraformaldehyde for 15 min, permeabilized with 0.1% Triton X‐100 (PBS) for 5 min, blocked with 1% (w/v) BSA (PBS) for 1 h, and then incubated with primary antibodies overnight. After washing, cells were incubated with fluorescence-labeled secondary antibody for 1 h at room temperature. The coverslips with cells were sealed with Fluor shield mounting medium with DAPI and imaged using a Super-Resolution Microscope (NIKON, N-SIM E). The number and size of p62 bodies were analyzed with the Image J software.

The primary antibodies were used: Smurf1 (Santa, sc100616; Abcam, ab57573), Keap1 (Proteintech, 10503-2-AP), Keap1 (Abcam, ab118285), p62 (Enzo, BML-PW9860), p62 (MBL, PM045), p62 (MBL, M162-3), LC3B (Sigma, L7543), LC3B (Novus, NB100-2220), NBR1 (Proteintech, 160004-1-AP), HA (MBL, M180-3), Nrf2 (Proteintech, 16396-1-AP), Flag (Sigma, F1804).

The secondary antibodies were used: Alexa Fluor^®^ 555 goat anti-mouse IgG (Life Technologies, A21425). Alexa Fluor^®^ 488 goat anti-rabbit IgG (Life technologies, A11008), Alexa Fluor^®^ 555 goat anti-rabbit IgG (Life technologies, A21428), Alexa Fluor^®^ 488 goat anti-mouse IgG (Life technologies, A11001), Alexa Fluor^®^ 647 goat anti-mouse IgG (Abcam, ab150115), Alexa Fluor^®^ 647 donkey anti-goat IgG (Abcam, ab150131), Alexa Fluor^®^ 405 goat anti-rabbit IgG (Abcam, ab175652).

### Preparing mouse embryonic fibroblasts (MEFs) from mouse embryos

Primary MEFs were obtained from embryos at 14-16 days post-coitum. The uterine horns of anesthetized pregnant mice were rinsed. The embryos were separated from placenta and placed in a 5 cm plate containing ice-cold sterile PBS. Remove the head, liver, and gut from the embryo and place the remaining portion in a 5 cm plate containing trypsin EDTA and cut into small pieces (~ 1 mm^3^) using a sterile razor blade and scissors. The chopped materials were incubated with 5 ml of trypsin EDTA for 30 min at 37 ℃ and then added 1 mL DMEM medium with 10% FBS to the chopped embryos, followed by centrifuging at 1000 rpm speed for 5 min. The pellets were resuspended and transferred to a 60 mm dish containing DMEM (with FBS and Penicillin/Streptomycin).

### Mice

*Smurf1*^+/+^, *Smurf1*^+/−^, and *Smurf1*^−/−^ mice were a kind gift from Dr. Lingqiang Zhang (Beijing Institute of Lifeomics, China). Mice were housed in specific pathogen‐free facilities, and the Ethics Review Committee for Animal Experimentation of Beijing Institute of Technology University approved the experimental protocol.

### Plasmids constructs

Full-length Smurf1 cDNA was amplified and then separately inserted into PKH3-3 × HA, 3 × Flag, pEGFP-N3, RFP and PGEX-5X-1 vector. Similarly, full-length p62 cDNA was amplified and then inserted into RFP-GFP, pEGFP-N3 and RFP vector. Full-length LC3 was amplified from RFP-GFP-LC3 (Addgene, #21074) and then inserted into PET-21a-Flag vector. Smurf1-C2, Smurf1-ΔC2, Smurf1-WW, Smurf1-HECT, Smurf1-ΔHECT was amplified from Flag-Smurf1 and then separately inserted into 3 × Flag. Flag-Smurf1-CS, Flag-Smurf1-ΔWW, Flag-Smurf1-CS-ΔWW, Flag-Smurf1^C699A^, GFP-p62^K7A/D69A^ and GFP-p62^S349A^ site mutant were generated by the site-directed mutagenesis. p62^K7A/D69A^ was amplified from GFP-p62^K7A/D69A^ and then inserted into RFP vector. Full-length Nrf2 was amplified from pCDNA3-Myc3-Nrf2 (Addgene, #21555) and then inserted into RFP vector. Full-length NBR1 was amplified from pMXs-IP GFP-NBR1 (Addgene, #38283) and then inserted into PKH3-3 × HA and pEGFP-N3 vector.

### Pull-down analysis

His-Flag, His-Flag-LC3, or GST-Smurf1 protein is extracted from E. coli strain BL21 using ProBond™ Purification System (Invitrogen, K85001) according to the manufacturer’s instructions. 20 μl of Flag beads (Sigma, A2220) were incubated with His-Flag or His-Flag-LC3 overnight at 4 °C and then washed with ice-cold sterile PBS three times. The beads were incubated with GST-Smurf1 for 6 h at 4 °C followed by washing with ice-cold PBS four times, then boiled in 2 × loading buffer (60 mM Tris-HCl, pH 6.8; 2% SDS; 0.1% Bromophenol blue; 25% Glycerol; 14.4 mM β-Mercaptoethanol) for 10 min and identified by western blot.

## Supplementary Information

**Additional file 1: Figure S1.** Smurf1 promotes the formation of p62-liquid droplets. **A** LN229 cells were transfected with Flag or Flag-Smurf1, fixed followed by immunofluorescence stained with anti-Smurf1 and anti-p62 antibodies. The nuclear was stained with DAPI. Bar: 5 µm. **B** LN229 cells with Flag, Flag-Smurf1, or Flag-Smurf1^C699A^ were fixed after treating with MG132 (20 µM, 12 h), then immunofluorescence stained with anti-p62 antibody. Bar: 5 µm. Bar graphs indicate the number and size of p62 puncta in each cell (mean ± SD, n = 10 cells examined over three independent experiments). The nuclear was stained by DAPI. NS* p* > 0.05, * p < 0.05, *** p < 0.001 as determined by unpaired two-tailed Student’s *t*-test. **C** LN229 cells cultured in glass-bottom plates were transfected with GFP-p62. Bar: 1 µm. The signal recovery after photobleaching was measured; mean ± SD, n = 20 droplets examined over three independent experiments. **D** LN229 cells cultured in glass-bottom plates were transfected with GFP-Smurf1 and treated with MG132 (20 µM, 12 h). Bar: 1 µm. The signal recovery after photobleaching was measured; mean ± SD, n = 20 droplets examined over three independent experiments. **Figure S2.** p62 phase separation is required for Smurf1-mediated Nrf2 activation. **A** LN229 cells were transfected with control or Smurf1 siRNA oligos. Cell lysates were prepared and subjected to western blot analysis with the indicated antibodies (anti-Nrf2, anti-p62, anti-Smurf1, and anti-β-actin). The right panels show relative intensity of p62 and Nrf2 in total cell (mean ± SD from 3 independent experiments). ** p < 0.01 as determined by unpaired two-tailed Student’s *t*-test. **B** 293T cells were transfected with control or Smurf1 siRNA oligos. Cytosolic and nuclear fractions were prepared and subjected to western blot analysis with the indicated antibodies (anti-Nrf2, anti-Smurf1, anti-H2B, and anti-β-actin). **C** LN229 cells were transfected with control or Smurf1 siRNA oligos. Total RNAs were prepared from these LN229 cells. The relative mRNA levels of Smurf1 and NQO1 were performed by qRT-PCR analysis. Values were normalized against the amount of mRNA in LN229 transfected with control siRNA oligos; mean ± SD from 3 independent experiments. *** p < 0.001 as determined by unpaired two-tailed Student’s *t*-test. **D** LN229 cells treated with control or p62 siRNA oligos were transfected with GFP, GFP-p62, or GFP-p62^K7A/D69A^. Cell lysates were prepared and subjected to western blot analysis with the indicated antibodies (anti-Nrf2, anti-GFP, anti-p62, and anti-β-actin). **Figure S3.** Smurf1 increases the binding affinity between p62 and Keap1 to activate Nrf2. **A** LN229 cells were transfected with control or Smurf1 siRNA oligos. Cell lysates were prepared and subjected to western blot analysis with the indicated antibodies (anti-p62, anti-p-p62^S349^, anti-p-p62^S403^, anti-Smurf1, and anti-β-actin). Bar graphs indicate the quantitative densitometric analysis of the indicated proteins relative to β-actin. Values were normalized against the intensity of LN229 transfected with si-Control; mean ± SD from 3 independent experiments. * p < 0.05, *** p < 0.001 as determined by unpaired two-tailed Student’s *t*-test. **B** LN229 cells treated with control or PTEN siRNA oligos were treated by treated by control or Smurf1 siRNA oligos. Cell lysates were prepared and subjected to western blot analysis with the indicated antibodies (anti-p-mTOR, anti-mTOR, anti-PTEN, anti-Smurf1, and anti-β-actin). **C** LN229 overexpressed with Flag or Flag-Smurf1 were transfected with HA or HA-PTEN, fixed after treating with MG132 (20 µM, 12 h) and then performed by immunofluorescence analysis with anti-p62 antibody. The nuclear was stained with DAPI. Bar: 5 µm. Bar graphs indicate the number and size of p62 puncta in each cell (mean ± SD, n = 10 cells examined over three independent experiments). NS *p* > 0.05, *** *p* < 0.001 as determined by unpaired two-tailed Student’s t-test. **D** LN229 cells were transfected with Flag or Flag-Smurf1, fixed after treating with MG132 (20 µM, 12 h), and then immunofluorescence stained with anti-Keap1 and anti-p62 antibodies. The nuclear was stained by DAPI. Bar: 5 µm. Bar graphs indicate the number of Keap^+^p62^+^ puncta in each cell (mean ± SD, n = 10 cells examined over three independent experiments). *** p < 0.001 as determined by unpaired two-tailed Student’s *t*-test. **E** LN229 cells overexpressed GFP-p62 were fixed after transfecting with Flag or Flag-Smurf1, and then immunofluorescence stained with anti-Keap1 and anti-Flag antibodies. The nuclear was stained with DAPI. Bar: 5 µm. **F** LN229 cells were transfected with control, Smurf1 or p62 siRNA oligos, fixed after treating with MG132 (20 µM, 12 h), and then immunofluorescence stained with anti-p62 and anti-Keap1 antibodies. The nuclear was stained by DAPI. Bar: 5 µm. Bar graphs indicate the number of Keap^+^p62^+^ puncta in each cell (mean ± SD, n = 10 cells examined over three independent experiments). NS* p* > 0.05, * p < 0.05 as determined by unpaired two-tailed Student’s *t*-test. **G** LN229 cells were transfected with control or p62 siRNA oligos, fixed after treating with MG132 (20 µM, 12 h), and then immunofluorescence stained with anti-Keap1, anti-p62, and anti-Smurf1 antibodies. The nuclear was stained by DAPI. Bar: 5 µm. **Figure S4.** NBR1 enhances the Smurf1 mediated p62 liquid-droplets accumulation. **A** LN229 cells were transfected with control or Smurf1 siRNA oligos. Cell lysates were prepared and subjected to western blot analysis with the indicated antibodies (anti-p62, anti-NBR1, anti-NDP52, anti-OPTN, anti-BAG3, anti-Smurf1, and anti-β-actin). Bar graphs indicate the quantitative densitometric analysis of the indicated proteins relative to β-actin. Values were normalized against the intensity of LN229 transfected with si-Control; mean ± SD from 3 independent experiments. NS* p* > 0.05, * p < 0.05, ** p < 0.01 as determined by unpaired two-tailed Student’s *t*-test. **B** LN229 cells treated with control or Smurf1 siRNA oligos were transfected with Flag or Flag-Smurf1, fixed after treating with MG132 (20 µM, 12 h), and then immunofluorescence stained with anti-NBR1 and anti-p62 antibodies. The nuclear was stained by DAPI. Bar: 5 µm. Bar graphs indicate the number and size of NBR1^+^p62^+^ puncta in each cell (mean ± SD, n = 10 cells examined over three independent experiments). * p < 0.05, ** p < 0.01 as determined by unpaired two-tailed Student’s *t*-test. **C** LN229 cells treated with GFP-p62 cultured in glass-bottom plates were transfected with control, Smurf1, or NBR1 siRNA oligos. The signal recovery after photobleaching was measured; mean ± SD, n = 20 droplets examined over three independent experiments. **Figure S5.** Smurf1 mediated NBR1 expression in p62 dependent manner **A** LN229 cells were transfected with control, Smurf1, p62 or NBR1 siRNA oligos. Cell lysates were prepared and subjected to western blot analysis with the indicated antibodies (anti-NBR1, anti-Smurf1, anti-p62, and anti-β-actin). **B** LN229 cells treated with control or Smurf1 siRNA oligos were transfected with Myc or Myc-Nrf2. Cell lysates were prepared and subjected to western blot analysis with the indicated antibodies (anti-Nrf2, anti-p62, anti-NBR1, anti-Smurf1, and anti-β-actin). **C** LN229 cells were treated with control or Smurf1 siRNA oligos and overexpressed Myc or Myc-Nrf2, fixed after treating with MG132 (20 µM, 12 h), and then immunofluorescence stained with anti-NBR1 and anti-p62 antibodies. The nuclear was stained by DAPI. Bar: 5 µm. Bar graphs indicate the number of NBR1^+^p62^+^ puncta in each cell (mean ± SD, n = 10 cells examined over three independent experiments). * p < 0.05, *** p < 0.001 as determined by unpaired two-tailed Student’s*t*-test. **D** LN229 cells treated with control, NBR1, Nrf2, or p62 siRNA oligos were transfected with HA or HA-NBR1, and then overexpressed Flag or Flag-Smurf1. Total RNAs were prepared from these LN229 cells. Bar graphs indicate the amount of NQO1 mRNA. Values were normalized against the amount of NQO1 mRNA in LN229 transfected with control siRNA oligos, HA, and Flag; mean ± SD from 3 independent experiments. NS* p* > 0.05, * p < 0.05, ** p < 0.01, *** p < 0.001 as determined by unpaired two-tailed Student’s *t*-test. **E** LN229 cells treated with NBR1 or p62 siRNA oligos were transfected with GFP, GFP-p62^WT^, GFP-p62^S349A^ or GFP-p62^K7A/D69A^, and overexpressed Flag, Flag-Smurf1, or HA-NBR1. Total RNAs were prepared from these LN229 cells. Bar graphs indicate the amount of NQO1 mRNA. Values were normalized against the amount of NQO1 mRNA in LN229 transfected with p62 siRNA oligos, HA-NBR1, GFP, and Flag; mean ± SD from 3 independent experiments. NS* p* > 0.05, * p < 0.05, ** p < 0.01, *** p < 0.001 as determined by unpaired two-tailed Student’s *t*-test. **Figure S6.** NBR1 enhances Smurf1-drived Nrf2-mediated oxidative stress response. **A** LN229 cells transfected with control or p62 siRNA oligos were overexpressed Flag or Flag-Smurf1, and treated with control (PBS) or H_2_O_2_ (200 µM, 2 h) after transfecting with control or NBR1 siRNA oligos. Cell lysates were prepared and subjected to western blot analysis with the indicated antibodies (anti-p62, anti-NBR1, anti-Flag, and anti-β-actin). **B** LN229 cells transfected with control or p62 siRNA oligos were overexpressed Flag or Flag-Smurf1, and treated with control (PBS) or H_2_O_2_ (200 µM, 2 h) after transfecting with control or NBR1 siRNA oligos. Total RNAs were prepared from these LN229 cells. Bar graphs indicate the amount of NQO1 mRNA. Values were normalized against the amount of mRNA in LN229 transfected with control siRNA oligos and Flag; mean ± SD from 3 independent experiments. NS* p* > 0.05, * p < 0.05, ** p < 0.01, *** p < 0.001 as determined by unpaired two-tailed Student’s *t*-test.

## Data Availability

All data that can support the conclusions of this article are included in the article. The cell lines and plasmids that were generated in this study will be available upon reasonable request. Further information and requests for resources and reagents should be directed to and will be fulfilled by the contact, Lei Dong (ldong@bit.edu.cn).
