# Supplementary material for: Enhanced liquidity of p62 droplets mediated by Smurf1 links Nrf2 activation and autophagy
Source: Cell Biosci. 2023 Feb 21;13:37. doi: 10.1186/s13578-023-00978-9 (PMC9945626; doi:10.1186/s13578-023-00978-9)

Additional Figure S1: Smurf1 promotes the formation of p62-liquid droplets

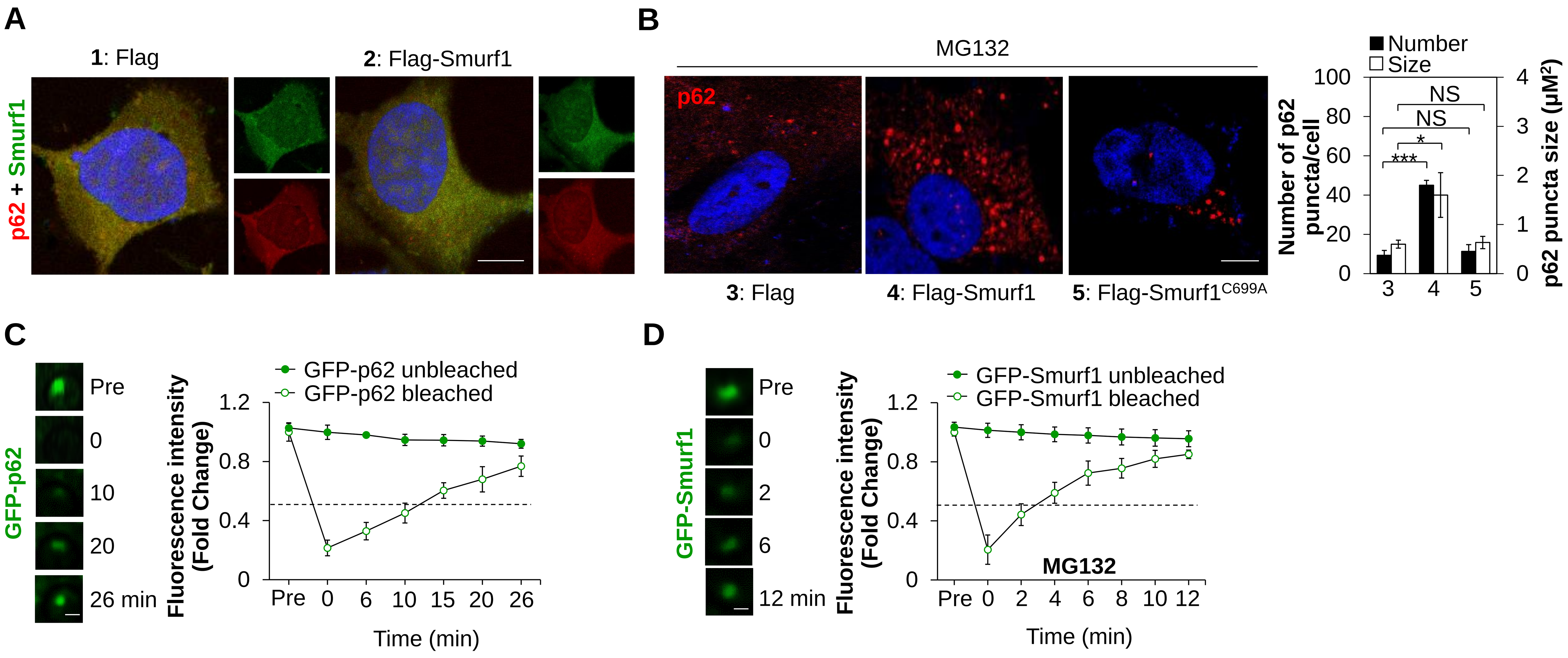

Additional Figure S2: p62 phase separation is required for Smurf1-mediated Nrf2 activation

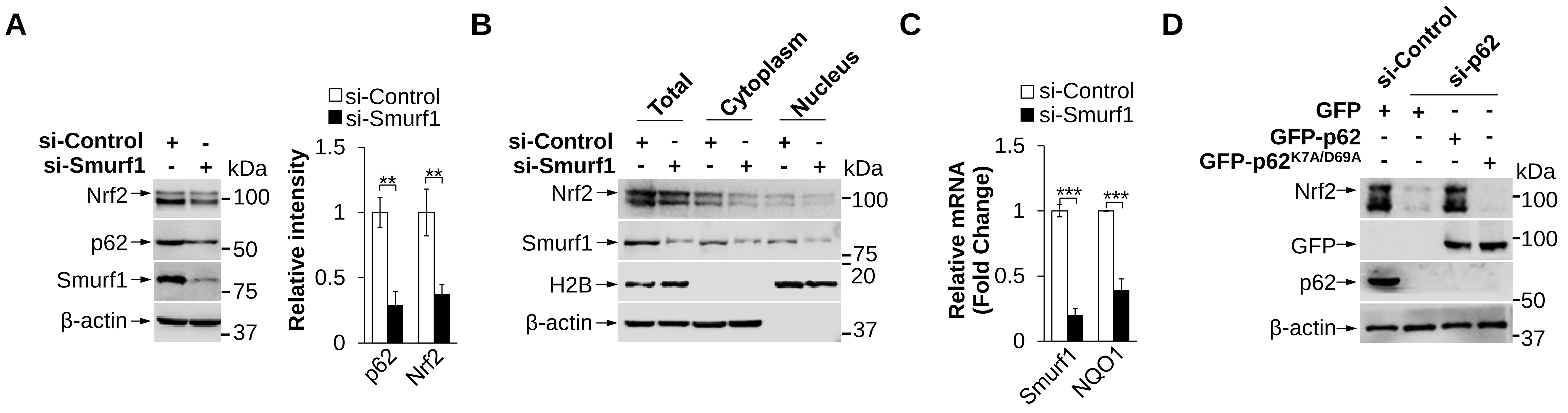

Additional Figure S3: Smurf1 increases the binding affinity between p62 and Keap1 to activate Nrf2

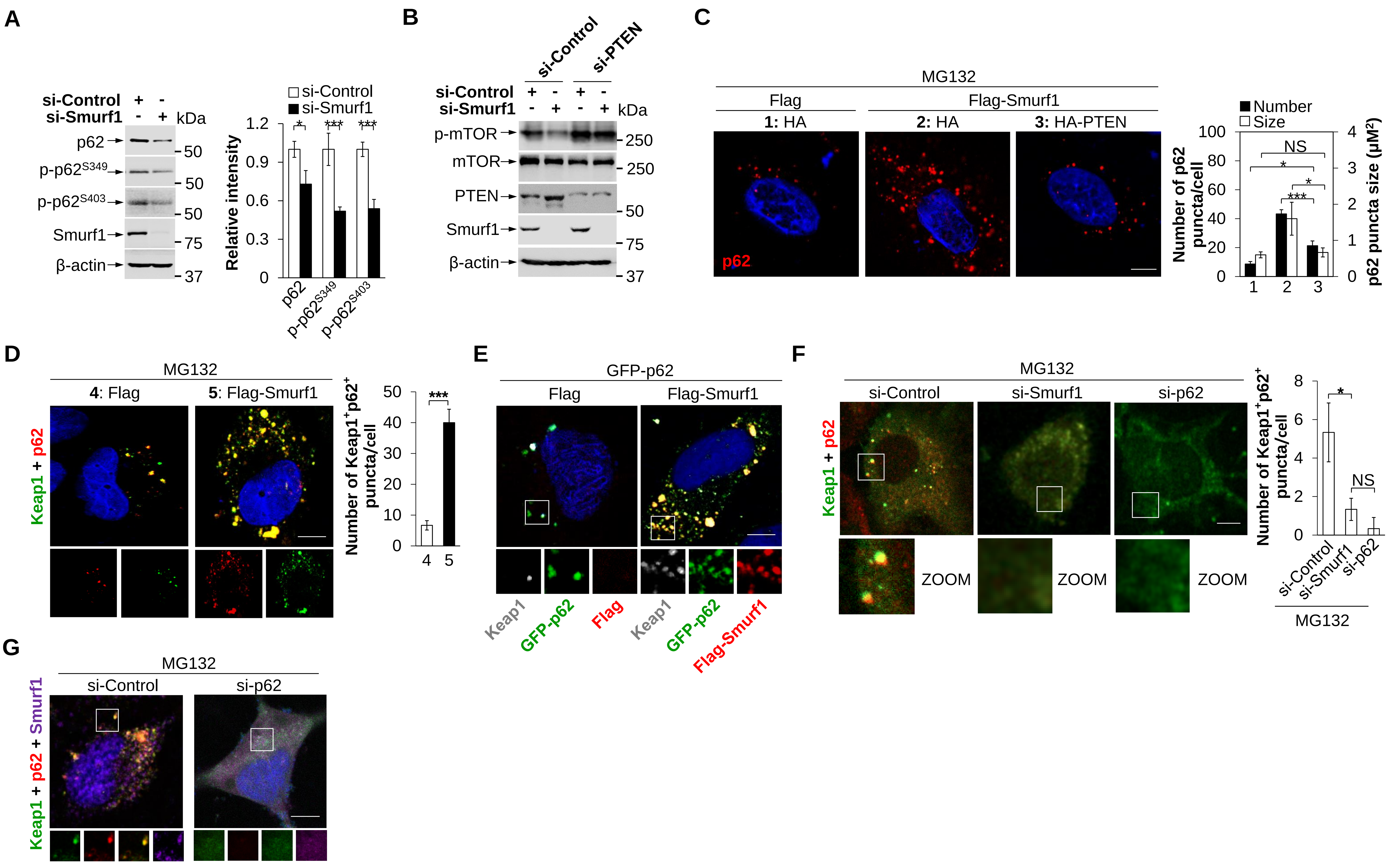

Additional Figure S4: NBR1 enhances the Smurf1 mediated p62 liquid-droplets accumulation

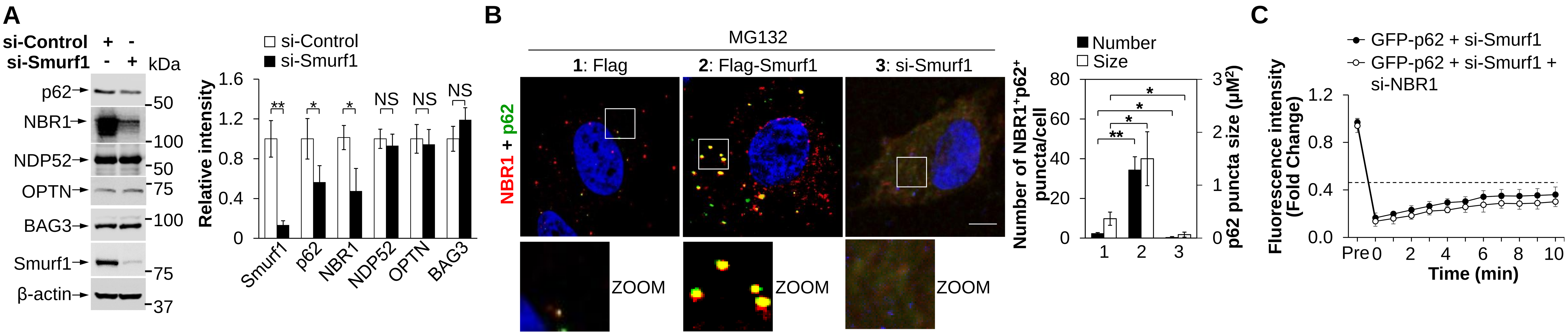

Additional Figure S5: Smurf1 mediated NBR1 expression in p62 dependent manner

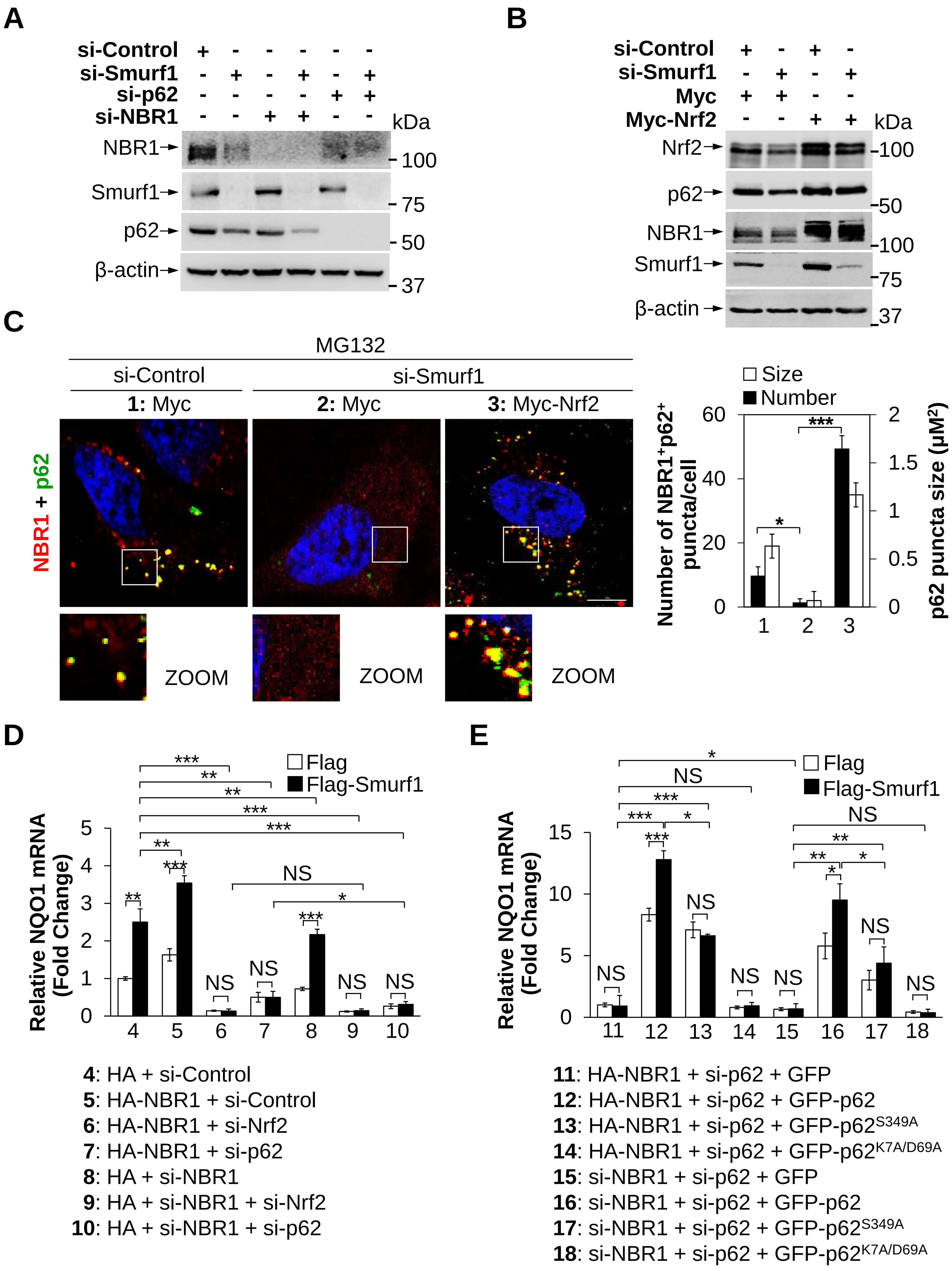

Additional Figure S6: NBR1 enhances Smurf1-drived Nrf2-mediated oxidative stress response

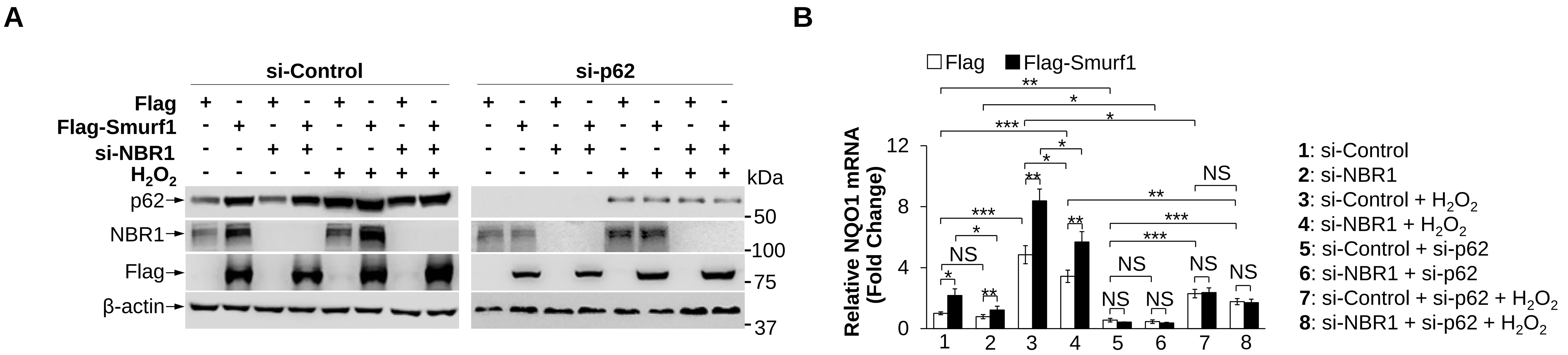

Supplement: Supplementary file 1 — Additional file 1: Figure S1. Smurf1 promotes the formation of p62-liquid droplets. A LN229 cells were transfected with Flag or Flag-Smurf1, fixed followed by immunofluorescence stained with anti-Smurf1 and anti-p62 antibodies. The nuclear was stained with DAPI. Bar: 5 µm. B LN229 cells with Flag, Flag-Smurf1, or Flag-Smurf1C699A were fixed after treating with MG132 (20 µM, 12 h), then immunofluorescence stained with anti-p62 antibody. Bar: 5 µm. Bar graphs indicate the number and size of p62 puncta in each cell (mean ± SD, n = 10 cells examined over three independent experiments). The nuclear was stained by DAPI. NS p > 0.05, * p < 0.05, *** p < 0.001 as determined by unpaired two-tailed Student’s t-test. C LN229 cells cultured in glass-bottom plates were transfected with GFP-p62. Bar: 1 µm. The signal recovery after photobleaching was measured; mean ± SD, n = 20 droplets examined over three independent experiments. D LN229 cells cultured in glass-bottom plates were transfected with GFP-Smurf1 and treated with MG132 (20 µM, 12 h). Bar: 1 µm. The signal recovery after photobleaching was measured; mean ± SD, n = 20 droplets examined over three independent experiments. Figure S2. p62 phase separation is required for Smurf1-mediated Nrf2 activation. A LN229 cells were transfected with control or Smurf1 siRNA oligos. Cell lysates were prepared and subjected to western blot analysis with the indicated antibodies (anti-Nrf2, anti-p62, anti-Smurf1, and anti-β-actin). The right panels show relative intensity of p62 and Nrf2 in total cell (mean ± SD from 3 independent experiments). ** p < 0.01 as determined by unpaired two-tailed Student’s t-test. B 293T cells were transfected with control or Smurf1 siRNA oligos. Cytosolic and nuclear fractions were prepared and subjected to western blot analysis with the indicated antibodies (anti-Nrf2, anti-Smurf1, anti-H2B, and anti-β-actin). C LN229 cells were transfected with control or Smurf1 siRNA oligos. Total RNAs were prepared from [file 13578_2023_978_MOESM1_ESM.pdf]
